# Reviving Élie Metschnikoff's *Monospora*: the obligately parasitic yeast *Australozyma monospora* sp. nov

**DOI:** 10.1093/femsyr/foaf041

**Published:** 2025-08-05

**Authors:** Marc-André Lachance, Carla E Cáceres, Molly J Fredericks, Meghan A Duffy, Tara E Stewart Merrill

**Affiliations:** Department of Biology, University of Western Ontario, London, Ontario N6A 5B7, Canada; School of Integrative Biology, University of Illinois Urbana-Champaign, Urbana, IL 61801, USA; School of Integrative Biology, University of Illinois Urbana-Champaign, Urbana, IL 61801, USA; Department of Ecology and Evolutionary Biology, University of Michigan, Ann Arbor, MI 48109, USA; Cary Institute of Ecosystem Studies, Millbrook, NY 12545, USA

**Keywords:** *Australozyma monospora* sp. nov, *Metschnikowia bicuspidata*, Daphnia spp, obligately parasitic yeast, asexual ascus formation

## Abstract

A vast literature explores a model system that consists of a prey crustacean, the water flea *Daphnia* spp., and an obligately pathogenic yeast that has been referred to as *Metschnikowia bicuspidata* and thought to represent the material used by Metschnikoff in his study of innate immunity. Typification of species bearing that name and indeed the whole genus has been problematic as regards yeasts that only grow or form aciculate ascospores *in hospite*. The neotype of *M. bicuspidata*, unlike the *Daphnia* parasite, is easily cultured on a variety of laboratory media, although it too can cause serious infections in a variety of mostly aquatic animals. It has become evident that the *Daphnia* parasite studied by Metschnikoff or current workers is not closely related to *M. bicuspidata* as currently understood. Analysis of whole genome DNA extracted from the yeast repeatedly found in infected *Daphnia* specimens shows that it belongs to the recently circumscribed genus *Australozyma*. The yeast is described here as *Australozyma monospora* sp. nov. The species, although haplontic and heterothallic, forms single-spored asci without mating. It also appears that all species in the genus are restricted to asexual reproduction, which may explain their rare status. The holotype is MICH 346683. The name is registered in Mycobank under the number MB 859667.

## Teaser

An obligate parasite of *Daphnia* spp. is described as *Australozyma monospora* sp. nov.

## Introduction

### Monospora

Élie Metschnikoff, in 1884, described the yeast species *Monospora bicuspidata*, which he illustrated in exquisite detail in the process of infecting water fleas (*Daphnia magna*). His images showed elongate cells, needle-shaped ascospores penetrating the gut lining of the animal, and phagocytic cells in the process of neutralizing the spores. He inferred that the spores were released from the asci by the action of the animal's gastric juices, later to give rise to elongate cells that divide by mostly terminal budding. The infection culminated in the extension of cells into acerose asci, each containing a single bicuspidate ascospore. Sensibly enough, he proposed the name *Monospora bicuspidata*. The yeast could not be cultured in laboratory media prepared from meat extract or fruit juice.

### Metschnikowia

Already in those early days of yeast systematics, complying with all requirements of the nomenclatural code applicable at the time (de Candolle 1867) was anything but a sinecure. Metschnikoff (1884) unfortunately was not aware that the name *Monospora* was already in use for an algal genus (Agardh 1876). This oversight was noticed by Kamienski (1899), who used the opportunity to pay tribute to Metschnikoff by renaming the genus in his honour and adding a second species, *M. artemiae*, an obligate parasite of brine shrimp. *Metschnikowia* prospered till 1913, when Genkel remarked that the name was also already in use and proposed the variant *Metschnikowiella* (Miller and van Uden 1970). Keilin (1920), evidently unaware of Kamienski's or Genkel's renamings, proposed the new genus *Monosporella* to accommodate *M. bicuspidata* as well as his newly discovered *Monosporella unicuspidata*, observed in diseased specimens of the midge *Dasyhelea obscura*. In addition to a different host, the new species formed ascospores that were pointed at only one end. Keilin's drawings were otherwise similar to Metschnikoff's. In spite of the two new names, *Monospora* was not yet in disuse, as evidenced by Rhuberg's (1933) observations on material collected on the German north coast. Of interest were drawings that featured, in addition to the figures presented by Metschnikoff (1884), ascospores with a large proximal or subdistal swelling.

### Metschnikowiella

Together with Chatton's Coccidiascus legeri (1913), yeasts forming single aciculate ascospores continued to be known exclusively from microscopic observations until 1961, when van Uden & Castelo-Branco described two free-living species, which they named *Metschnikowiella krissii* and *Metschnikowiella zobellii*. At the time, yeast identification and species delineation were based primarily on morphology plus a few growth test responses that in the present case were used to distinguish the two species. The morphologies were similar, however. On V8 agar sporulation medium, the thallus consisted of typical ovoid, multilaterally budding cells and the asci were ovopedunculate. The authors also conducted experimental infections of *Daphnia magna*. Their drawings of cells and asci observed in infected material remarkably resembled Metschnikoff's (1884). Specifically, the cells were elongate and formed terminal, elongate buds on a broad base; the asci were acerose, bearing no vestiges of the ovoid parent cells, unlike what was seen on V8 agar. All drawings indicated the presence of a single ascospore per ascus, as did the written description. Soon after, van Uden (1962) took the determining step of reinstating the genus *Metschnikowia*, upon realizing that Genkel's (1913) rejection of the name was unnecessary, as the name's previous application was to a sponge species and therefore not considered a homonym under the botanical code. This notwithstanding, *Metschnikowiella* was the name used by Green (1974) in a review of parasites and epibionts of cladocerans. *Daphnia* specimens collected in the London region contained organisms similar to those reported by Metschnikoff (1884), and a novel species, *M. dianae*, was described based on longer asci from material collected in Ecuador.

### Rejection of *Monospora*

The addition of growth test responses as taxonomic descriptors complicated the matter of typification. Having discovered two species with nearly identical morphologies, van Uden and Castelo-Branco (1961) remarked that it had become impossible to determine whether Metschnikoff's (1884) material consisted of only a single species, leading them to reject *Monospora bicuspidata* as a *nomen dubium*. A similar action was taken by Spencer et al. (1964) upon describing *Metschnikowia kamienskii* (now considered a synonym of *Metschnikowia bicuspidata*), which was isolated from *Artemia salina*, Kamienski's source of isolation for *M. artemiae*. Again, as living material was not available for the latter, it was not possible to establish its boundaries as a kinship-based species.

### Neotypification

The discovery of free-living species assigned to *Metschnikowia* engendered the problem of linking living types to those based on fixed material or even drawings, both of which continue to be allowed by the Code of Nomenclature (Turland et al. 2018). Wickerham (1964) carefully assessed the issue and proposed for *Metschnikowia* a living neotype. The material had been recovered from a parasitic trematode, *Diplostomum flexicaudum*, found in the digestive gland of the snail *Stagnicola emarginata angulata* in a Michigan lake. Wickerham thought that the morphology of the free-living strain was sufficiently similar to that of Metschnikoff's (1884) material to be used as a substitute, although he fell short of declaring that they were indubitably conspecific. With the caveat that it was impossible to equate Wickerham's free-living neotype to Metschnikoff's drawings, Miller and van Uden (1970) accepted his neotypification, which remains in force today (Lachance 2011). Indeed, new developments provided direct evidence that the two are unlikely to be conspecific. Miller et al. (1967), upon digesting asci of *M. krissii* and *M. zobellii* with cell envelope lytic enzymes, demonstrated in the latter the presence of two ascospores instead of one. It soon became evident (Miller and van Uden 1970) that most known free-living *Metschnikowia* species form two ascospores, although asci with only one or no spore at all may occur as part of normal variation. In closely related, free-living *Metschnikowia* species capable of hybridization, the number of spores does vary somewhat, but the formation of two ascospores by most asci, in crosses between compatible mating types, is indicative of fertility, whereas the formation of mostly single-spored or empty asci indicates hybrid sterility (Lachance and Bowles 2004, Lee et al. 2020).

### Challenges to the free-living neotype

Wickerham's (1964) recruitment of a type, not only for *Metschnikowia bicuspidata*, but also for the entire genus, did not put the matter to rest. Codreanu and Codreanu-Balcesku (1981), having observed asci in *Daphnia magna* and *Artemia salina* in Romania, argued that they had rediscovered the original *M. bicuspidata* and *M. artemiae*, respectively. Unlike Metschnikoff (1884) or Kamienski (1889), they provided light micrographs of the “sporiferous” asci. Unfortunately, the images are of insufficient resolution to allow one to discern ascospores. Electron micrographs did support the presence of a single ascospore per ascus, but lacked the context needed to evaluate morphology holistically. The written descriptions specified that the ascospores of the two species were bicuspidate and 38–56 and 45-58 μm long, respectively, for *M. bicuspidata* and *M. artemiae*. As Wickerham's (1964) proposed neotype of *M. bicuspidata* was isolated from a trematode, in Michigan, it was unlikely to represent the same species as that found in *Artemia salina* by Kamienski (1899), because of differences in geography, hosts, and ascospore numbers. This view was held also by Weiser et al. (2003), who further proposed, presciently as it happens, that free-living and obligately parasitic species should be assigned to separate genera. Nonetheless, they considered recognizing, as genuine *Metschnikowia* species, Metschnikoff's *M. bicuspidata*, Kamienski's *M. artemiae*, Keilin's *M. unicuspidata*, and importantly, Wickerham's neotype, renamed *M. wickerhami*, as proposed by Codreanu and Codreanu-Balcesku (1981). They also added the description, based on morphology, of *M. typographi*, to accommodate an obligately parasitic species found in two species of bark beetles (*Ips* spp.) collected in two European localities. The new species formed acerose asci, often with a central swelling similar to that reported by Rhuberg (1933) and seen also in some large-spored *Metschnikowia* species (*e.g. M. mauinuiana*, Lachance 2011). Again, however, the asci of *M. typographi* were documented with light micrographs of low resolution, insufficient to show the spores (Codreanu and Codreanu-Balcesku 1981). Here, electron micrographs demonstrated the presence of two ascospores, such that an anticipated genus of obligate parasites would be polymorphic for ascospore numbers. Morphologically similar specimens were observed in the great spruce bark beetle *Dendroctonus micans* in Turkey (Yaman and Radek 2008). Although too low to allow one to discern ascospores, the resolution of the light micrographs was sufficient to show that the asci were similar in shape to those observed by Weiser et al. (2003). The same can be said of micrographs provided by Kleespies et al. (2017) for similar material, but these authors took the major step of amplifying DNA from yeast-rich samples, successfully determining a barcode sequence with a high nucleotide identity to those of free-living *Metschnikowia agaves* and *Metschnikowia* (*Candida*) *wancherniae*, neither of which is a close relative to *M. bicuspidata. M. agaves* was known exclusively from necroses of tequila agave plants in Mexico (Lachance 1993) until it was isolated from papaya skin (Gu et al. 2013) and used in the fermentation of pineapple wine (Lin et al. 2022). *M. wancherniae* is known from plant surfaces in Thailand (Nakase et al. 2009). This encouraging development must be tempered by the caveat that the coincident observation of asci and the amplification of a particular DNA sequence in the same sample do not guarantee that the amplified DNA originated from the asci. Other corroborative evidence remains necessary.

### Conservation of *Metschnikowia*

One can now be confident that the current usage of the name *Metschnikowia* is as permanent as anything can be in taxonomic nomenclature. For this we owe a debt of gratitude to Alexander B. Doweld, who in 2015, convinced the Nomenclature Committee for Fungi to conserve the name and that of the family Metschnikowiaceae against competing designations. This is all the better, given that the genus has now grown to include over sixty described species, some of which, including *M. bicuspidata*, are of considerable socioeconomic importance. The Linnean binomial *Metschnikowia bicuspidata* is “forever” attached to Wickerham's (1964) neotype, and congeneric species will bear the name *Metschnikowia* for the foreseeable future. The extent of the genus is another matter, as will soon become apparent.

### Australozyma

The genus *Australozyma* Q.M. Wang, Yurkov, Boekhout & F.Y. Bai originated in 2024 when Liu et al. sought to clarify the classification of genera within the family Metschnikowiaceae by phylogenomics. Many species included in the family had been originally assigned to the genus *Candida*, a polyphyletic form-genus that faithfully, and for many years, fulfilled the important duty of hosting ascomycetous yeast species of uncertain affinity due to the absence of a known sexual life cycle. With the advent of DNA sequencing, phylogenetically based generic assignments became the norm, and species originally ascribed to *Candida* are gradually being reassigned to more meaningful genera, such as *Australozyma*. As there currently is no monograph of the genus, it is appropriate here to give a short account. The eight described species form a well-supported clade in phylogenomic trees (Liu et al. 2024, Opulente et al. 2024), where the genus constitutes a sister clade to *Metschnikowia* as currently understood. If one accepts the axiom that a genus should be monophyletic, an argument for treating the two as a single genus remains tenable. Best practices dictate that there should be compelling reasons to separate them. One must be vigilant as, for reasons difficult to explain, there has been a repeatedly expressed yearning to see *Metschnikowia* split into smaller fragments, starting with the publication of its first sequence-based phylogeny by Mendonça-Hagler et al. (1993), who suggested that *M. hawaiien*sis and possibly *M. lunata* should be removed. A similar view was advocated somewhat forcefully by Kurtzman et al. (2018), who felt that *Metschnikowia* in fact consists of several genera. The authors even left out the entire large-spored species clade (Lachance 2011) in their analysis of the genus. The prospect of fragmenting *Metschnikowia* was also brought up, but thankfully not acted upon, by Liu et al. (2024).

*Australozyma picinguabensis* (Ruivo et al.) Q.M. Wang, Yurkov, Boekhout & F.Y. Bai, *comb. nov*. and *Australozyma saopauloensis* (Ruivo et al.) Q.M. Wang, Yurkov, Boekhout & F.Y. Bai, *comb. nov*. were described as *Candida* species by Ruivo et al. (2006) from material recovered in water accumulating in the flower bracts of *Heliconia velloziana* in the Brazilian Atlantic Forest. Each is represented by a small number of isolates. A short digression relevant to the vagaries of systematics is appropriate. The epithet *saopauloensis* was originally proposed as currently spelled, but the nomenclature experts of the journal required that it be modified to *saupaulonensis*. This was later treated as an orthographic error by other experts, who restored the original spelling. *Australozyma bambusicola* (Nakase et al.) Q.M. Wang, Yurkov, Boekhout & F.Y. Bai, *comb. nov., Australozyma nongkhaiensis* (Nakase et al.) Q.M. Wang, Yurkov, Boekhout & F.Y. Bai, *comb. nov., Australozyma succicola* (Nakase et al.) Q.M. Wang, Yurkov, Boekhout & F.Y. Bai, *comb. nov*. (type species), and *Australozyma touchengensis* (Nakase et al.) Q.M. Wang, Yurkov, Boekhout & F.Y. Bai, *comb. nov*. were described simultaneously by Nakase et al. (2011), also as *Candida* species, based on single isolates from, respectively, insect frass of bamboo, insect frass of an unidentified plant, and an exudate of *Ficus racemosa*, all in Thailand, as well as soil from Taiwan. The authors made a strong argument for describing these species from singletons as they are rarely encountered otherwise. *Australozyma robnettiae* (M. Groenew. et al.) Q.M. Wang, Yurkov, Boekhout & F.Y. Bai, *comb. nov*. was one of five unrelated *Candida* species described by Groenewald et al. (2011). It was one of four single isolates obtained by enrichment from the surface of unspecified flowers in Guyana. The species is a sister to *Candida tocantinsensis*, which was described from two isolates recovered in substrates similar to those of *A. picinguabensis* and *A. saopauloensis* (Barbosa et al. 2012). The kinship with *A. robnettiae* was not known at the time, but a divergence of six substitutions in the D1/D2 LSU rRNA gene sequence attests to their close relatedness. A genome sequence is not available, such that the species was not formally included in *Australozyma* by Liu et al. (2024), nor will it be considered further here. *Australozyma saccharicola* Kaewwich. ex Q.M. Wang, Yurkov, Boekhout & F.Y. Bai, *sp. nov*. was described as *Metschnikowia saccharicola* by Kaewwichian et al. (2012). Four strains were recovered by enrichment from sugarcane leaves in Thailand. The growth test responses of the eight species are typical of those encountered in many *Metschnikowia* species, but with two notable exceptions. N-acetyl glucosamine utilization, which generally gives a strong response in *Metschnikowia* species (as both a carbon and a nitrogen source), is not assimilated. Unlike *Metschnikowia* species, *Australozyma* species generally grow well in the presence of 0.01% cycloheximide. Both differences can be regarded as sufficient synapomorphies to justify separation of the two genera. Given that the first species description only goes back to 2006 (Ruivo et al.), it was reasonable for Nakase et al. (2011) to conclude that rarity also is a common trait of the genus. Of the eight described *Australozyma* species, the literature contains only two reports of new encounters. Tan et al. (2018) examined an isolate of *A*. (*M*.) *saccharicola*, recovered from a marine sediment, for the production of a killer toxin capable of inhibiting the growth of *M. bicuspidata*, an important pathogen of an industrially reared crab in China. Most recently, *A*. (*C*.) *saopauloensis* was identified as the agent of an infection in an infant (Ning et al. 2024). More reports are to be expected as the GenBank database features deposits for eight distinct isolations for *A. saopauloensis*, four for *A. picinguabensis*, and three for *A. saccharicola*. In addition, barcode sequences are available for a novel, close relative of the first two species, and two other novel species of less certain placement within the genus.

### Pathogenic Metschnikowiaceae

Infections of a wide range of animals by *M. bicuspidata* have been reported. A recent review (Hansali et al. 2023) lists among victims the Chinese mitten crab, brine shrimp, the ridgetail white prawn, chinook salmon, giant freshwater prawn, the marine crab, the mud crab, the mangrove land crab, the Chinese grass shrimp, sea urchins, and waterfleas (*Daphnia* spp.). There is even a report (Moore and Strom 2003) of Chinook salmon dying of yeast septicemia after feeding on infected brine shrimp. In that instance, the identity of *M. bicuspidata* was ascertained by barcode DNA sequencing, but that has not always been the case, notably when the suspected agent cannot be cultured *ex hospite*. Application of the barcode sequence approach to the subject at hand began in 2009, when Wolinska et al. amplified DNA from multiple samples of infected water fleas from European lakes, comparing them with material received from colleagues in the United States. The sequencing results revealed two important facts, namely that there was little variation among both European and North American samples, and, surprisingly, that the yeast is clearly not *M. bicuspidata*, but some other species showing only moderate relatedness to any known species of the family Metschnikowiaceae. This is particularly important as the *Daphnia*-yeast system is the object of considerable activity in modelling host-parasite interactions (e.g. Ebert 2005, Duffy and Sivars‐Becker 2007, Cáceres et al. 2009, Hall et al. 2009, Strauss et al. 2015, Lopez and Duffy 2021, Stewart Merrill et al. 2021, Ebert 2022). In all cases, the parasite is said to be *Metschnikowia bicuspidata*, based on its resemblance to the yeast studied by Metschnikoff in *Daphnia*. However, as discussed above, identity of the yeast that is currently being studied in *Daphnia* to the one studied by Metschnikoff cannot be established with certainty. What is now clear is that the yeast that has been the subject of considerable study in *Daphnia* is not the same species as the *M. bicuspidata* neotype.

Searle et al. (2015) conducted a barcoding study on infected *Daphnia* recovered in American lakes, confirming the presence of yeast DNA similar to that found in Europe by Wolinska et al. (2009); this was expected, as Wolinska et al. had also included samples of infected *Daphnia* collected from three lakes in the United States, with two lakes in common between the two studies. Other barcode sequences are accumulating in GenBank (C.L. Shaw, T.Y. James, M.A. Duffy, unpublished). Arhendt et al. (2018) sequenced the genomes of eight highly diverse parasitic fungi that have so far resisted *in vitro* culturing. Among these were extracts of infected water fleas. A broad fungal phylogeny positioned the yeast within the Ascomycota, nearest to an authentic strain of *M. bicuspidata*, although the two were recognized as different. Most interestingly, the authors examined all genomes for possible clues as to the parasites’ inability to grow on laboratory media. Disparate fungi had in common deficiencies in the metabolism of polyamines, sulfate reduction, biotin, and thiamine. The *Daphnia* parasite, whose genome was labelled Baker2002 in the NCBI database, lacked genes for 15 enzymes present in *M. bicuspidata*. These included components of thiamine synthesis, although this may not be so remarkable given that many yeasts require one to several vitamins for their growth. However, the genes for two enzymes involved in the thiamine pathway were absent in the parasite but present in authentic *M. bicuspidata*. Genes for the central metabolism enzymes citrate synthase and ATP citrate synthase were also reported to be lacking in the parasite. Jiang et al. (2022) compared the genomes of various strains identified as *M. bicuspidata*, including Baker2002, in the search for insights into the genomics of pathogenicity. Among other things, they observed that genes accounting for carbohydrate-active enzymes, or so-called CAZymes, tended to be lower in yeast species with known pathogenicity. The Baker2002 genome ranked the lowest, with *ca*. 117 CAZymes, compared to 133 in a free-living *M. bicuspidata*, and 173 in the ubiquitous saprobe, *Debaryomyces hansenii*. Most relevant to the present discussion, the authors showed that Baker2002 has no detectable gene collinearity with authentic *Metschnikowia* species, pathogenic or not.

The loss of *in vitro* culturability that accompanies pathogenicity is likely to occur gradually, such that it might be possible to observe instances where the process is underway but not complete. The so-called arizonensis clade of *Metschnikowia* is named after its first-discovered member, *M. arizonensis*, reported by Lachance and Bowles (2002) to exhibit poor viability when grown on laboratory media. This and some other clade members lose vigor once in culture, to the extent that some of the original isolates of *M. arizonensis* were lost before they could be preserved cryogenically. The species was isolated on multiple occasions from nitidulid beetles in flowers of a single cactus plant, despite attempts to recover it from neighboring specimens. The beetles had a lethargic demeanor, suggesting the presence of a pathology. Whether the species represents a case of host dependence *in statu nascendi* remains to be explored.

### Objectives

This study aims at clarifying the classification and nomenclature of the parasitic yeast that serves as a major model system for the study of host-parasite interactions involving *Daphnia* species. The species, to be named *Australozyma monospora* sp. nov. is almost certainly the same as that observed by Metschnikoff in 1884 and which he named *Monospora bicuspidata*, although in the absence of type material for the latter, this cannot be demonstrated beyond a reasonable doubt.

## Materials and methods

The work described here was performed on cultures maintained by successive infection of *Daphnia dentifera* with yeast ascospores originally recovered from Baker Lake in Barry County, Michigan, in 2002. The crustacean was fed every other day with the alga *Ankistrodesmus falcatus*. Phase contrast microscopy was performed on material held between a thin agar slab and a coverslip, with a Leitz Ortholux phase contrast microscope. *In situ* images of cells or asci were obtained by differential interference microscopy (DIC) with an Olympus BX53 microscope with a Nomarski prism and DP73 camera, or by scanning electron microscopy following the methods in Rodrigues et al. (2008); briefly, samples were dehydrated in ethanol, dried, mounted, coated with gold, then imaged with a JEOL 6300F field emission scanning electron microscope at the Michigan State University Center for Advanced Microscopy. Phase contrast images were recorded with an Amscope MU1000 camera. Some made use of the focus stacking feature of the provided software, and others were taken separately at different focal planes and later merged with the *focusstackingonline* application. Images were enhanced for brightness and contrast with the *Photos* application included with Microsoft Windows 11.

Cultures of two free-living *Australozyma* species, *A. picinguabensis* UNESP 00–89 and *A. saopauloensis* UNESP 00–99, were obtained from the University of Western Ontario yeast collection, where they are kept in liquid nitrogen. Attempts to obtain ascus formation entailed mixing cultures on various sporulation media, namely YM, Yeast Carbon Base with 0.01% yeast extract (YCBY), malt extract (1 and 5%), and V8 (1/5 and 1/20), all solidified with 1.5% agar.

Genome sequences were obtained from NCBI or from the National Microbiology Data Center. Accession numbers are given in the supplemental data online, Table S1. Genomes were selected to represent the various clades identified in the family Metschnikowiaceae by Liu et al. (2024), including all eight described species of *Australozyma*. Every clade within the large genus *Metschnikowia* was represented by one or more species. Orthologous genes used to construct a robust phylogeny were recovered using the Geneious Prime platform in the following manner. Scaffolds of the Baker2002 genome were queried, starting with the largest, against the genome of the outgroup *Debaryomyces hansenii* (Debaryomycetaceae) to increase the likelihood that hits with closer relatives would be sufficiently conserved across the entire spectrum of species to provide useful phylogenetic signals. Baker2002 gene sequences with hits of 1000 bp or larger were then queried by BLASTn against a local database of all genomes under consideration. The resulting hits were evaluated for orthology, aligned with MAFFT (Katoh and Standley 2013), and spliced when fragmented. Only genes with clear orthologs in all species were retained. For each gene, a maximum likelihood phylogeny was assembled using FastTree with the GTR model (Price et al. 2009), as implemented in Geneious Prime. Each tree was examined for potential anomalies and retained if none were detected. As the survey progressed, a concatenation of accumulated sequences was used to infer a tree and verify the robustness of the topology as indicated by values of the Shimodaira-Hasegawa test (1999). The 108 selected genes are listed in the supplementary data (Table S2) along with their alignment size and the placement of Baker2002 suggested by each individual tree. They were recovered from the first 10 scaffolds of the genome plus gene RCY1, which was examined in the characterization of the mating locus. The final species tree was obtained with the RaXml (Stamakis 2014) plugin of Geneious Prime, with the GTR model and 100 bootstrap replicates, after removal of gapped and invariant positions. Characterization of genes associated with mating employed information gathered for *Clavispora lusitaniae* by Reedy et al. (2009) and for many *Metschnikowia* species by Lee et al. (2018). Assessment of synteny made use of the Emboss dotter dotplot (Madeira et al. 2024) feature of Geneious Prime. The word size was adjusted to maximize the signal/noise ratio as required. The program OAT, version 093.1 (Lee et al. 2015), was used to calculate OrthoANI.

## Results and discussion

### Phylogeny

**Figure 1.**
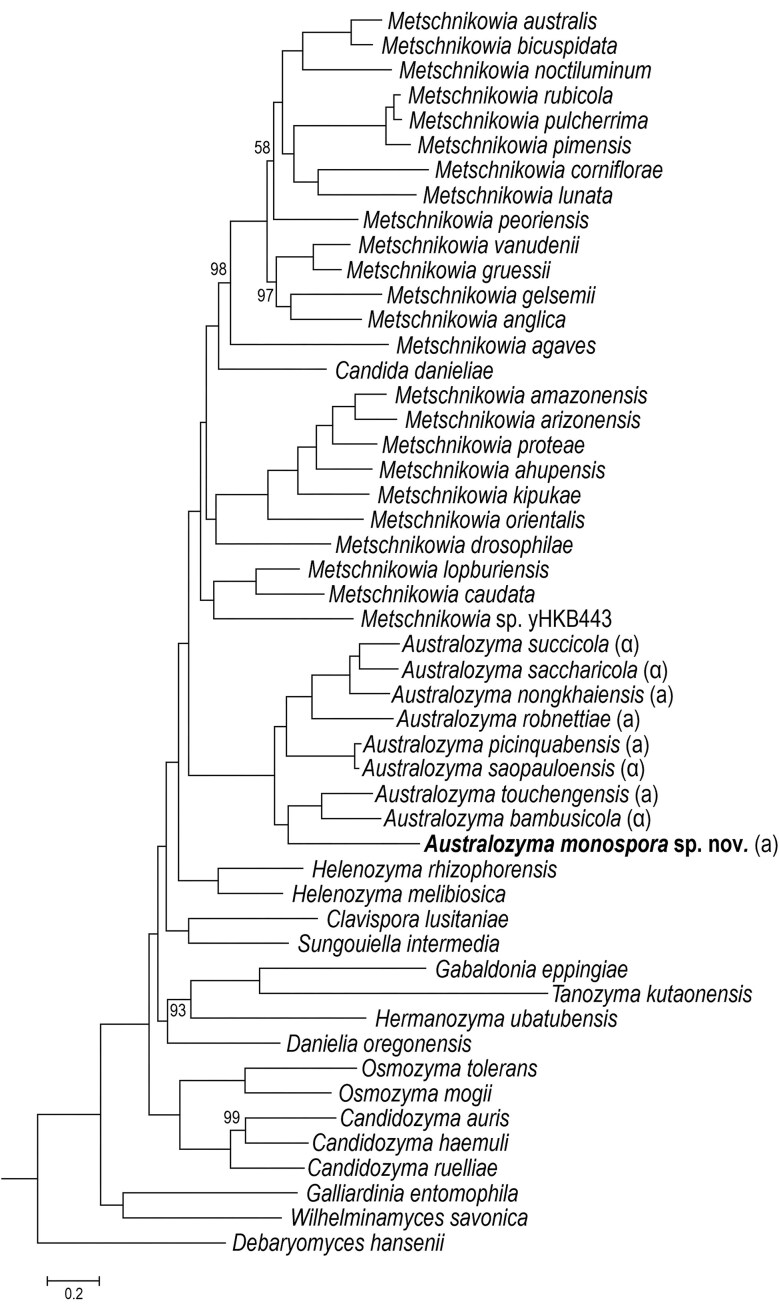
Phylogeny of selected Metschnikowiaceae species, showing the position of *Australozyma monospora* sp. nov. Baker2002, based on a concatenation of 108 protein-coding genes and inferred with RAxML using the GTR model, following removal of gapped and invariant positions. Bootstrap values for 100 iterations are shown only when less than 100%. *Debaryomyces hansenii* (Debaryomycetaceae) was used as outgroup to root the tree.

Alignments of 108 genes were assembled in a concatenation of 111 564 informative positions and used to infer a species tree (Fig. 1). Inspection of individual trees revealed that the majority (88) supported membership of Baker2002 (*A. monospora* sp. nov.) in the genus *Australozyma* (Table S2). Among these, 42 positioned the species in an early-diverging position, as did a species tree obtained by neighbor-joining or FastTree (not shown). Alignments of 33 genes placed it as a sister to the *A. bambusicola-A. touchengensis* pair, as did the species tree (Fig. 1), and 13 suggested other placements within *Australozyma*. Twenty placements fell in various positions outside the genus. Outgroup taxa that paired with Baker2002 were species with low confidence positions, including seven cases of pairing with *Tanozyma kutaoensis*, a representative of a monotypic genus (Yuan et al. 2012) that sits at the tip of a long, isolated branch in Fig. 1 and in other genome-based phylogenies (Liu et al. 2024, Opulente et al. 2024). The pairing is likely due to long-branch attraction, as no evidence was found for horizontal gene transfer between the two. Specifically, the genes associated with unusual placements were not clustered or located in local islands of synteny. Importantly, membership of Baker2002 in *Australozyma* is strongly supported in the species tree, as evidenced by the long subtending internode (the longest in the entire tree), attesting to the integrity of the clade.

The phylogeny in Fig. 1 agrees well with those obtained by Liu et al. (2024) and Opulente et al. (2024), despite their different taxon sampling intensities. Here, a preliminary examination of average nucleotide identity (OrthoANI) values had indicated that the Baker2002 sequence might join the *Australozyma* clade, for which reason all genomes available for the genus were included in the analysis. As the matter at hand also included assessing the possibility that the *Daphnia* parasite might be better assigned to *Metschnikowia*, that genus was represented by one or more members of each subclade within the genus. The approach used here in assembling the dataset differs in the manual selection of genes, which may reduce the chance of misinterpreting paralogs for orthologs; according to Moody et al. (2022) incorrect identification of orthologs can be a major source of inaccuracies in reconstructing deeper nodes from alignments generated automatically by common algorithms. The selection of genes based on high sequence identity with the selected outgroup favored the retention of conserved, as opposed to rapidly evolving, genes. It is worth noting that the general placement of the various genera obtained by neighbor-joining agreed more with that of RAxML compared to that of FastTree, which did not resolve *Metschnikowia* as a single sister clade to *Australozyma*, as suggested by all other analyses (NJ and FastTree results not shown).

### Microscopy

**Figure 2.**
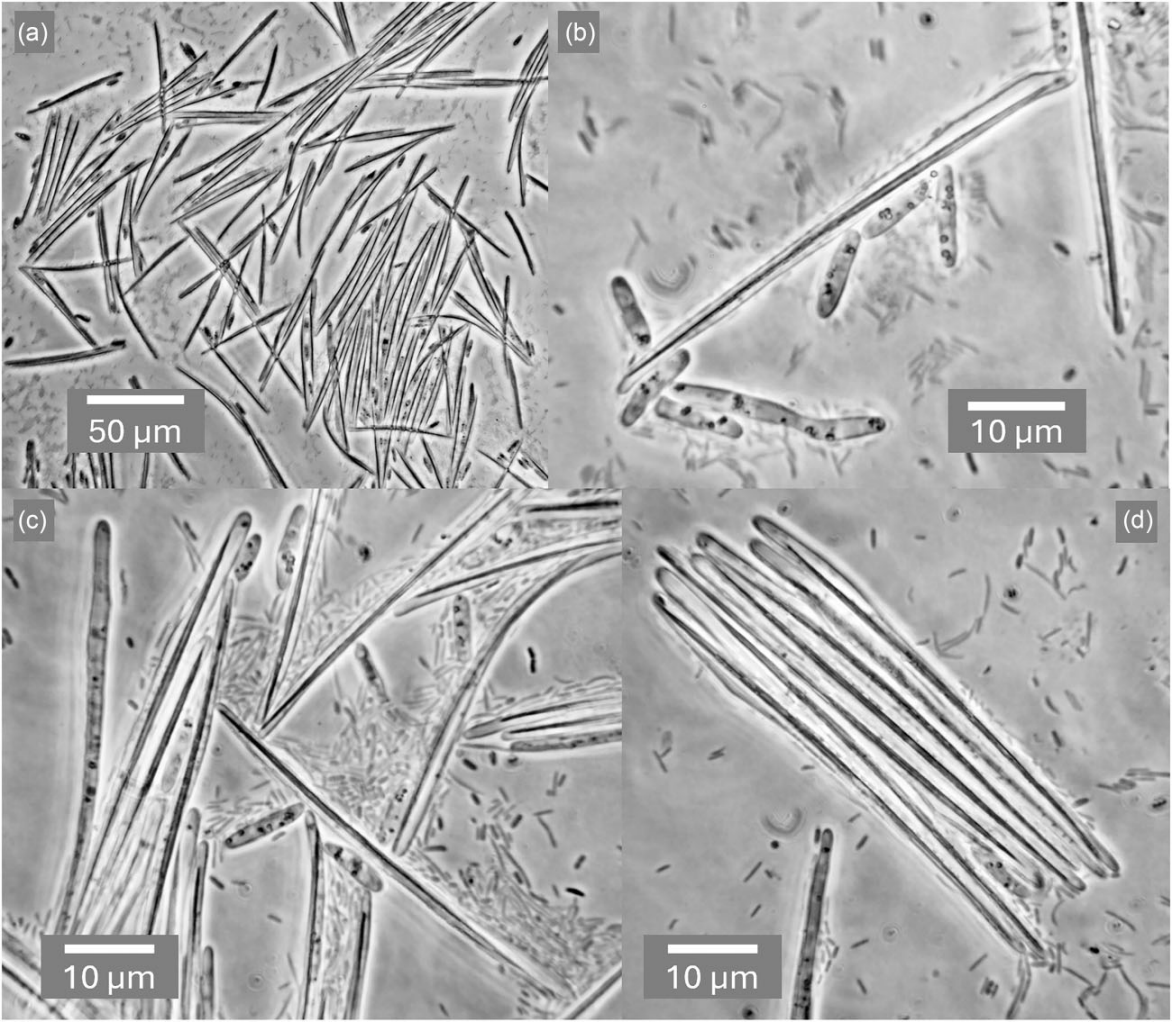
Asci and cells of *Australozyma monospora* sp. nov. extruded from diseased *Daphnia dentifera* two weeks post-infection. (a) Dense cluster of mature asci. (b) Elongate cells and asci. (c) Immature (left) and mature asci with cells. (d) A cluster of mature asci, clearly showing the presence of a single aciculate ascospore in each ascus. Bacteria are visible in the background. Phase contrast. Panel a is from a single image with a 20x objective. Panels b, c, and d are stacks, each merged from two images captured at different focal planes with a 100x objective.

**Figure 3.**
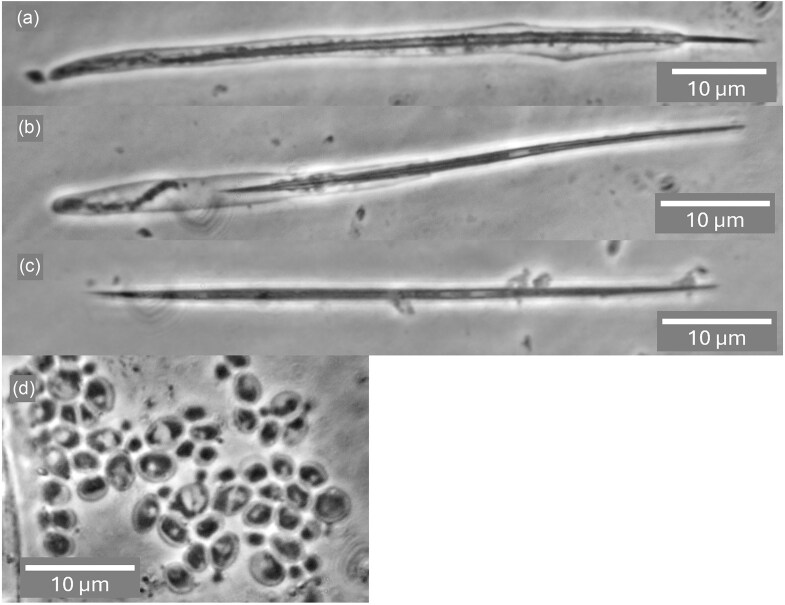
*Australozyma monospora* sp. nov. at early stages of the infectious cycle (5 days post-infection). Ascospore release by the action of the enzymes of *Daphnia dentifera* (a,b,c). Budding cells of the actively growing yeast (d). All images are stacks of 2–3 phase contrast micrographs at different focal planes with a 100x objective.

Well over a century after Metschnikoff's (1884) description of the time course of infection of *Daphnia magna* by *Monospora bicuspidata*, Stewart Merrill and Cáceres (2018) documented the phenomenon with light micrographs, referring to the yeast as *Metschnikowia bicuspidata*. Their images show free aciculate ascospores in the animal's gut, some of which transpierce the gut lining while being met by adhering haemocytes. After two to four days, protuberances emanating from spores begin the invasive phase, eventually giving rise to dense clusters of globose cells that the authors described as conidia enclosed in a sporocyst, although a common envelope is not easily perceived. By six to eight days, these give way to elongate budding cells that proliferate abundantly, and by nine to ten days morph into masses of acerose structures that are undoubtedly asci, although spores cannot be distinguished. The images presented here in Figs. 2 and 3 (a-c) are comparable in resolution to Metschnikoff's (1884) hand drawings and clearly confirm his rendition of both asci and cells. Panel a of Fig. 2 shows asci photographed at low magnification, but where the resolution and contrast allow one to see ascospores, a feature that is regrettably lacking in hitherto published light micrographs depicting obligately parasitic yeasts. Higher magnification images (Fig. 2, b–d; Fig. 3, a–c) show the consistent presence of a single ascospore per mature ascus. Fig. 2 (b,c) also features elongate cells with an aspect ratio of 5:1 or more, similar to those observed by Metschnikoff (1884). These bear some resemblance to the pseudohyphal cells shown in Fig. 5, panel b, which might suggest that the host environment may favour the more invasive, pseudohyphal growth habit, as recognized in vertebrate pathogens such as *Candida albicans* (Chen et al. 2020).

**Figure 4.**
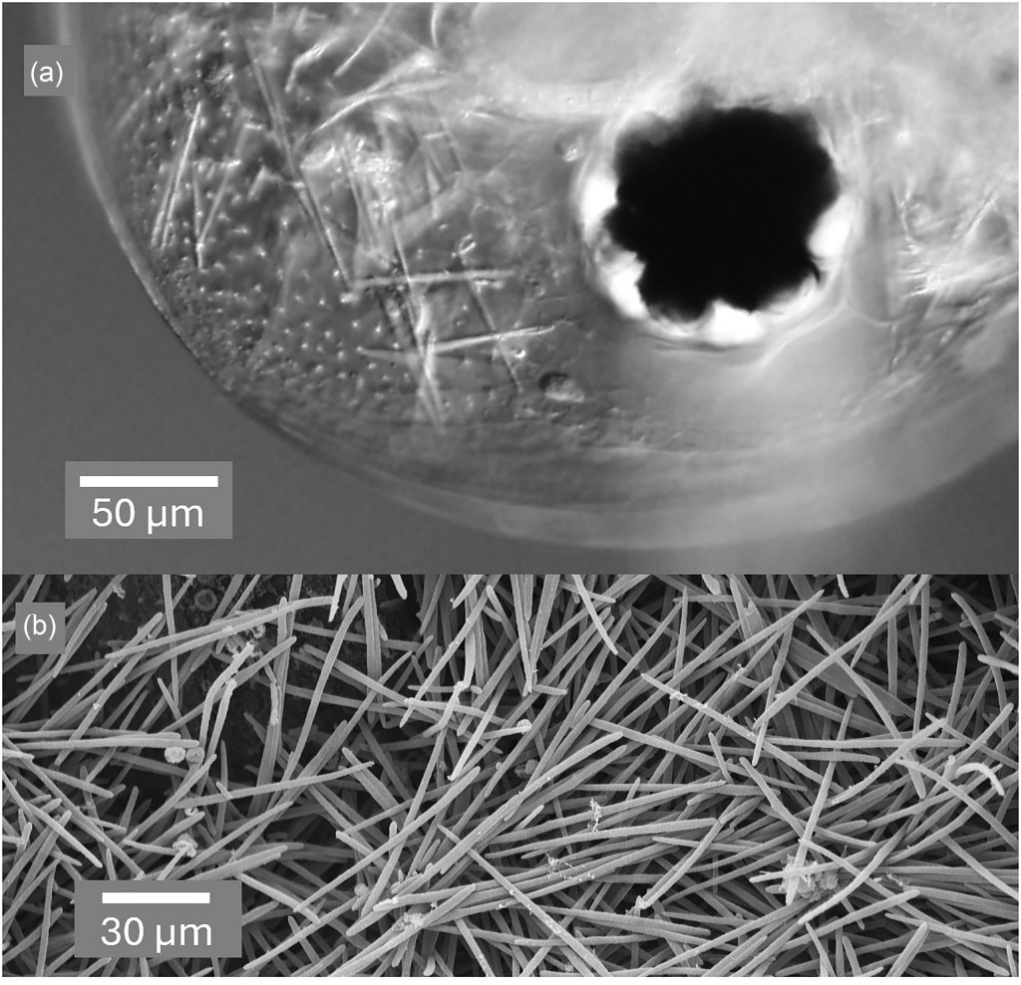
*In situ* images of *Australozyma monospora* sp. nov. in infected *Daphnia dentifera*. (a) Intact asci are visible by DIC next to the eye in the transparent head and (b) by SEM in the perforated body.

Once in contact with the digestive enzymes of a new host, asci are dissolved, releasing free ascospores (Fig. 3, a–c). Budding cells typical of known free-living *Australozyma* species (Ruivo et al. 2006, Nakase et al. 2011, Kaewwichian et al. 2012) also develop (Fig. 3d) and may account for the sporocyst conidia described by Stewart-Merrill and Cáceres (2018). As the infection progresses, the body cavity of the host fills with asci (Fig. 4), which upon release are ready to repeat the cycle.

### Mating loci

The Metschnikowiaceae examined so far (Reedy et al. 2009, Riley et al. 2016, Lee et al. 2018) share a common mating system, with two complex alleles, or idiomorphs, that determine mating compatibility (Fig. 6). The *MAT****a*** locus contains two mating genes, *MTa1*and *MTa2*, that code for transcription factors that initiate the expression of genes involved in the mating process in mating type **a** strains. The *MAT****α*** version contains the single *MT****α****1* gene, responsible for triggering expression of the mating type **α** features. The loci also contain three genes that are not directly involved in sexual reproduction. Termed non-sex genes, *PIK1, PAP1*, and *OBP1* are not collinear between the two idiomorphs (Fig. 6), but their order within each mating type is conserved across the Metschnikowiaceae. This prevents meiotic recombination between idiomorphs, such that the three non-sex genes constitute paralogs and not orthologs. Hence, separate phylogenies of non-sex genes of mating type **a** or **α** loci both reflect the ancestry of the species, but a combined phylogeny of the two sets of genes (Lee et al. 2018) shows them to have diverged with the common ancestor of the order Serinales, which includes both the families Metschnikowiaceae and Debaryomycetaceae (Groenewald et al. 2023). In principle, the sequence of any non-sex gene can be used to identify the mating type (**a** or **α**) of any species in the Serinales, as was done here for *Australozyma* species (see mating type assignments in Fig. 1). The genes directly responsible for the mating phenotype itself, which includes the mating pheromones and their receptor proteins, are unlinked to the mating loci, which only serve as activators. It is important to remain aware that individuals of either mating type, determined by the unique *MAT* locus, possess all genes required to generate both **a** and **α** pheromones and their receptors.

The genome analysis suggested that *Australozyma* species are haplontic and heterothallic, which implies that their eventual life cycle would be expected to begin with the conjugation of cells of complementary mating types, resulting in a zygote that matures into an ascus. In most haplontic *Metschnikowia* species (*M. agaves* and below in Fig. 1), conjugation begins within 4 hours after contact; asci reach full size after a day and spore maturity is observed after two days. In the Metschnikowiaceae, one would expect the ascus to contain two ascospores, and not four, as anticipated in archetypal yeasts such as the model species *Saccharomyces cerevisiae*. The *MAT***a** and *MAT****α*** loci are distributed equally (5:4) among *Australozyma* species, each of which is documented from only one genome sequence, except for *A. saopauloensis*, whose two independent isolates have the same mating idiomorph, *MAT****α***. The genomes of the two strains are nearly identical, as evidenced by an OrthoANI value of 99.0%. For reasons that will become apparent, it remains to be seen whether further sampling will identify both mating types in any of the species. The Baker2002 genome features a *MAT***a** idiomorph typical of those of other mating type **a**  *Australozyma* species.

The estimated length of the *MAT* loci in the genus ranged from 6.3 to 9.0 kb, and the extent of synteny surrounding the mating loci varied as a function of relatedness (Table S3), from as little as 1.2 kb between more distant species, to over 200 kb for close relatives. As for all Metschnikowiaceae species examined thus far, the mating loci of *Australozyma* species are flanked upstream by the *MAS2* orthologous gene (Fig. 6), whose phylogeny mirrors that of the species and not that of the genes of the mating locus. The *MAS2* gene thus defines the break point beyond which mating type loci are protected from recombination. *Australozyma* species are exceptional, however, in that the downstream flank does not feature such a clear boundary. Other Metschnikowiaceae (Reedy et al. 2009) and even some other members of the Serinales (Riley et al. 2016) punctuate the downstream end with an obscure open reading frame labeled “orf19.3202”, followed by gene *RCY1*. In *Australozyma* spp., *RCY1* is unlinked to the mating type locus, and attempts to recover *3202* homologs did not bear fruit. In fact, the downstream region lacks immediate conserved sequences that could be used to infer the exact point where paralogy gives way to orthology. This is even more remarkable in the light of the fact that *3202* and *RCY1* also sit adjacent to the mating locus in *D. hansenii*, a representative of the Debaryomycetaceae (Fig 6). The unlinked *RCY1* gene in itself could be regarded as a synapomorphy that justifies the separation of *Australozyma* from *Metschnikowia*.

Further anomalies include the relocation outside the mating locus of the *PAP1* gene in both *A. bambusicola* (mating type **α**) and *A. touchengensis* (mating type **a**), causing the two sister species to feature nearly collinear mating loci, although the remaining non-sex genes show no signs of having homogenized by recombination. Not so for the *PAP1* gene, which is relocated in another, large syntenic region, as are the *RCY1* genes, although the two are unlinked. Indeed, the *PAP1* gene of *A. touchengensis* (mating type **a**), clusters with mating type **α** homologs (data not shown), consistent with the loss of protection against recombination in a still sexually competent ancestral lineage that led to the two extant species. To our knowledge, this is the first documented example, in the Metschnikowiaceae, of a variant arrangement of non-sex genes.

All *Australozyma* species possess conserved regions that align in part with the published positions of the *MTa1, MTa2*, or *MTα1* transcription factor genes of other Metschnikowiaceae, but open reading frames could not be identified with confidence. It is important to note that identifying these genes, even in *Metschnikowia* species known to undergo a normal sexual cycle, is not straightforward.

Summing up, features of the *MAT* loci of *Australozyma*, especially the lack of a clear downstream boundary and the relocation of the *PAP1* gene in two species, one of each mating type, raise the suspicion that the mating locus may no longer be functional and that the ability to mate and hence to undergo karyogamy and meiosis may be lacking in some or perhaps all species. The failure to recover both complementary mating types in isolates of any one *Australozyma* species may therefore be more than a sampling intensity problem. It will be of interest to screen independent samples of infected *Daphnia* for amplification of mating type-specific sequences to see if the species represented by Baker2002 consists entirely of lineages with the same mating type (**a**). As things now stand, a reasonable prediction is that it will.

### Mating pheromone genes

Mating pheromone peptides of Metschnikowiaceae are typically encoded in a manner that enhances their transcriptional yield, but by two distinct modalities. The loci responsible for the **a**-pheromone (*MF***a**) are often found in multiple copies, whereas **α**-pheromones are encoded by a single, polycistronic locus. This was the case among *Australozyma* species, where the number of **a**-pheromone loci varied from one (*A. bambusicola*) to four, although these translated into no more than two distinct peptides per species (Fig. 7). In addition to two versions that had the potential to be functional, Baker2002 (*A. monospora* sp. nov.) had two pseudogenes, as evidenced by the absence of anything resembling the CVIA motif that serves as target of farnesylation of the **a**-pheromone, as documented in *S. cerevisiae* (Michaelis and Barrowman 2012). The motif is conserved in the Metschnikowiaceae examined by Lee et al. (2018). It is not clear whether the predominance of CVIV or CTIV motifs (Fig. 7) in *Australozyma* is of any consequence. Ashok et al. (2020) found that some variants of the CVIA motif do allow peptide prenylation in *S. cerevisiae*. Another intriguing feature is the high degree of sequence variation in the **a**-pheromone peptide (past the vertical line in Fig. 7) observed across *Australozyma* species. In contrast, haplontic *Metschnikowia* species had nearly identical **a**-pheromone peptide sequences (Lee et al. 2018), their mating compatibility being determined primarily by the **α**-pheromones.

The genomes of all eight free-living *Australozyma* species contained multiple versions of the **α**-pheromone coding sequences, arranged in tandem repeats of five or six copies (not shown), except for *A. robnettiae*, where only a triplet was observed. The total numbers of unique nucleotide and peptide sequences were, respectively, 31 and eight (Fig. 8). The variation in peptide diversity is comparable to that encountered among the **α**-pheromones of *Metschnikowia* species (Lee et al. 2018) and follows phylogenetic lines (not shown). Most noteworthy here is the absence of an *MF****α*** gene in Baker2002. To find out whether this might be due to missing portions of the genome resulting from sequencing artefacts, the gene with flanking sequences was queried against the Baker2002 genome. The search yielded a large (*ca*. 40-80 kb depending on the queried species) syntenic background as elicited by dotplot analysis. A short sequence with minimal identity with *MF****α*** genes could be identified where the gene should have been expected. This observation adds considerable weight to the notion that *A. monospora* sp. nov. has lost the ability to engage in sexual reproduction.

### Other genes involved in sexual reproduction

Mating pheromones act by being secreted and binding to membrane-bound receptor proteins of compatible partners. The receptors are encoded by genes *STE2* (expressed in **α**-cells) and *STE3* (expressed in **a**-cells). The genes *IME2, SPO11*, and *STE12* are also known to play vital roles in both mating and meiosis in *Clavispora lusitaniae* (Sherwood et al. 2014), a Metschnikowiaceae species. Ostensibly functional copies of all five genes were identified in all *Australozyma* species genomes, including Baker2002. The persistence of these genes suggests that some of the processes involved in sexual reproduction, such as ascogenesis in *A. monospora*, are active in these yeasts, although they may have been repurposed.

### Hybridization

**Figure 5.**
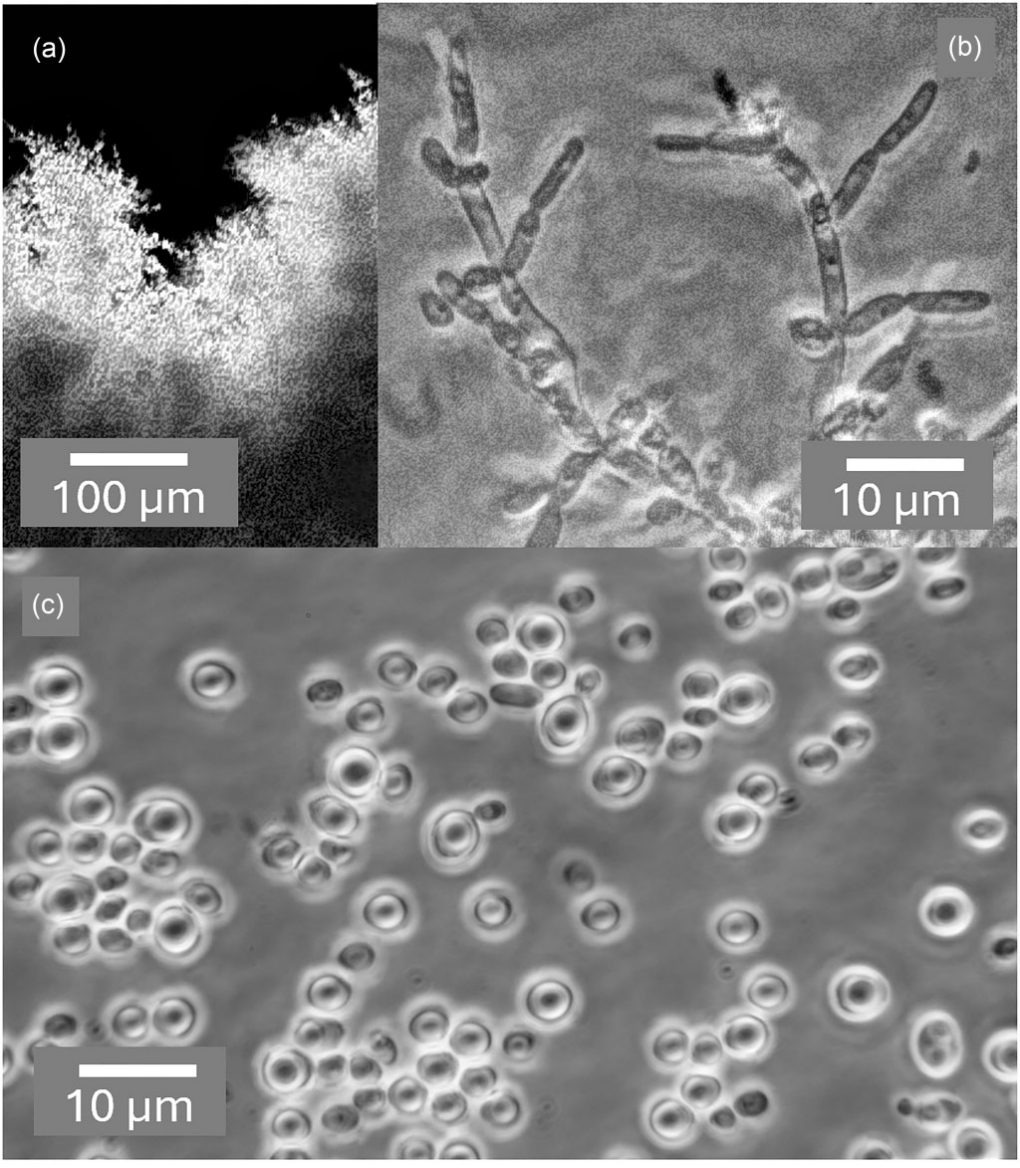
Growth of a mixed culture of *Australozyma picinguabensis* UNESP 00–89 and *A. saopauloensis* UNESP 00–99. (a) Submerged pseudohyphae in agar, (b) pseudohyphae near the agar surface, and (c) budding cells; YCBY agar after 5 days at room temperature. Traces of conjugation or ascogenesis are lacking. Phase contrast. Panel a is from a single image with a 10x objective. Panel b is a continuous stack captured with the Amscope software; panel c is from a stack of three merged images; both with a 100x objective.

The above considerations notwithstanding, it would be negligent to dismiss the possibility of hybrid matings. The sister taxa *A. picinguabensis* and *A. saopauloensis* are closely related. An OrthANI value of 95.7% could even be interpreted as evidence for conspecificity, although the 18 substitutions in the D1/D2 LSU rRNA gene sequences argue otherwise (Ruivo et al. 2006). The two species produce identical **α**- and **a**-pheromones and are known from strains of complementary mating types. Hence, they stood the best chance of mating when co-cultured. Equivalent pairs of *Metschnikowia* species invariably mate and give rise to zygotes and asci, but not necessarily ascospores (Lee et al. 2018, Lachance et al. 2020). The relevant cross was performed with the two type strains, using a wide variety of sporulation media, but no signs of mating were detected (Fig. 5).

**Figure 6.**
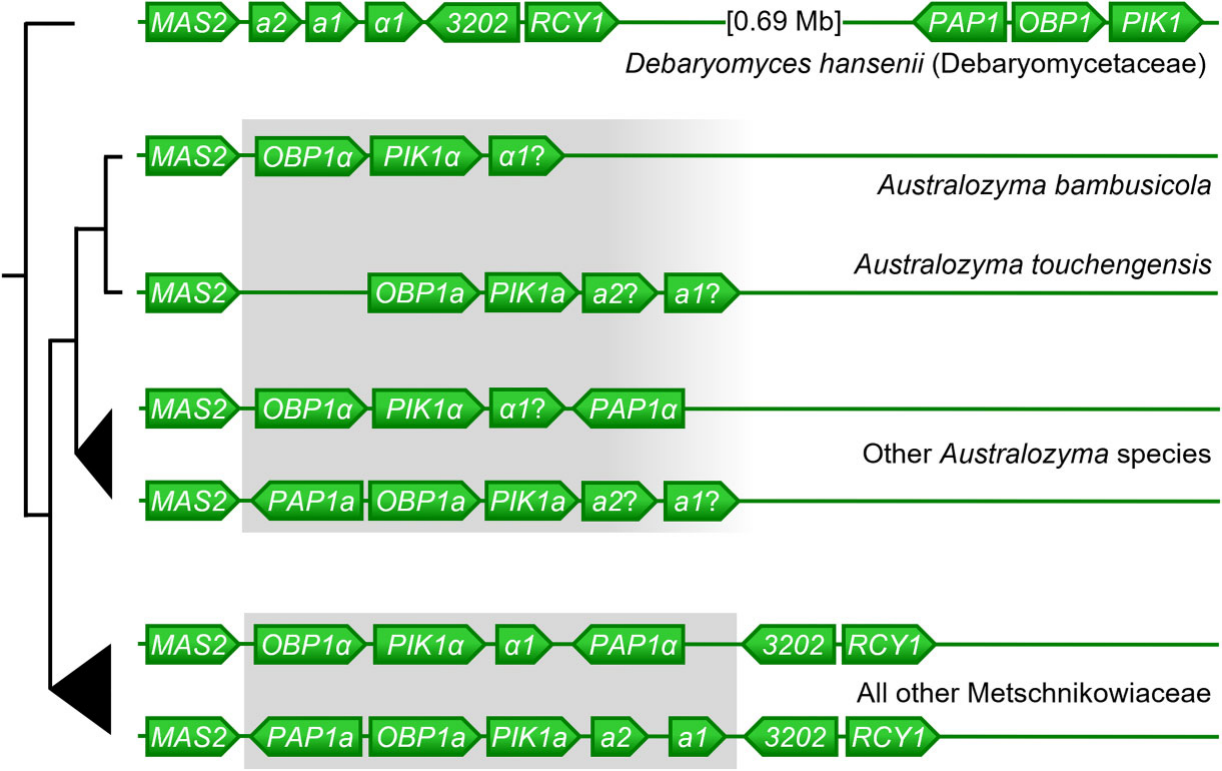
Overview of the mating type loci (*MAT***a** and *MAT****α***) in *Australozyma* spp., all other Metschnikowiaceae, and *Debaryomyces hansenii* (Debaryomycetaceae), modified from Lee et al. 2018). Shading identifies the mating locus proper in the Metschnikowiaceae. The downstream boundary in *Australozyma* is undefined, as are potential mating type genes *MT***a** and *MT****α***, abbreviated as a*1*, a*2*, and *α1*. The non-sex genes *PAP1, PIK1*, and *OBP1* are paralogs specific to each mating type. The *PAP1* genes are unlinked to the *mat* locus in two species. The scales are approximate.

**Figure 7.**
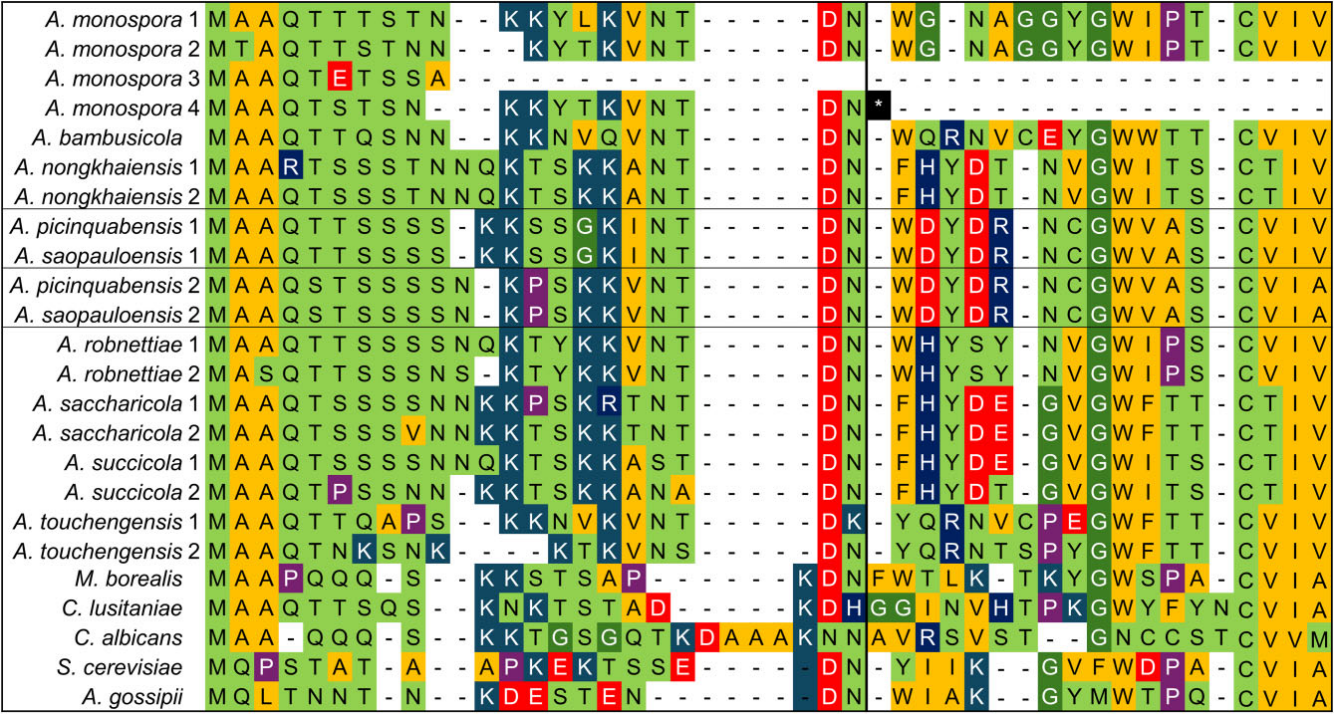
Amino acid alignment of **a**-pheromone open reading frames of *Australozyma* species. The sequence of the pheromone itself begins at the vertical line. Identical sequences for pairs of sister species are boxed. In *A. monospora* sp. nov., two sequences represent pseudogenes: version 4 features a stop codon approximately midway; in version 3, there were no alignable nucleotides past the position corresponding to the tenth amino acid. Sequences for five outgroup species are reproduced from Fig. 6 of Lee et al. (2018). The colours follow those generated by Geneious Prime to reflect amino acid polarity.

**Figure 8.**
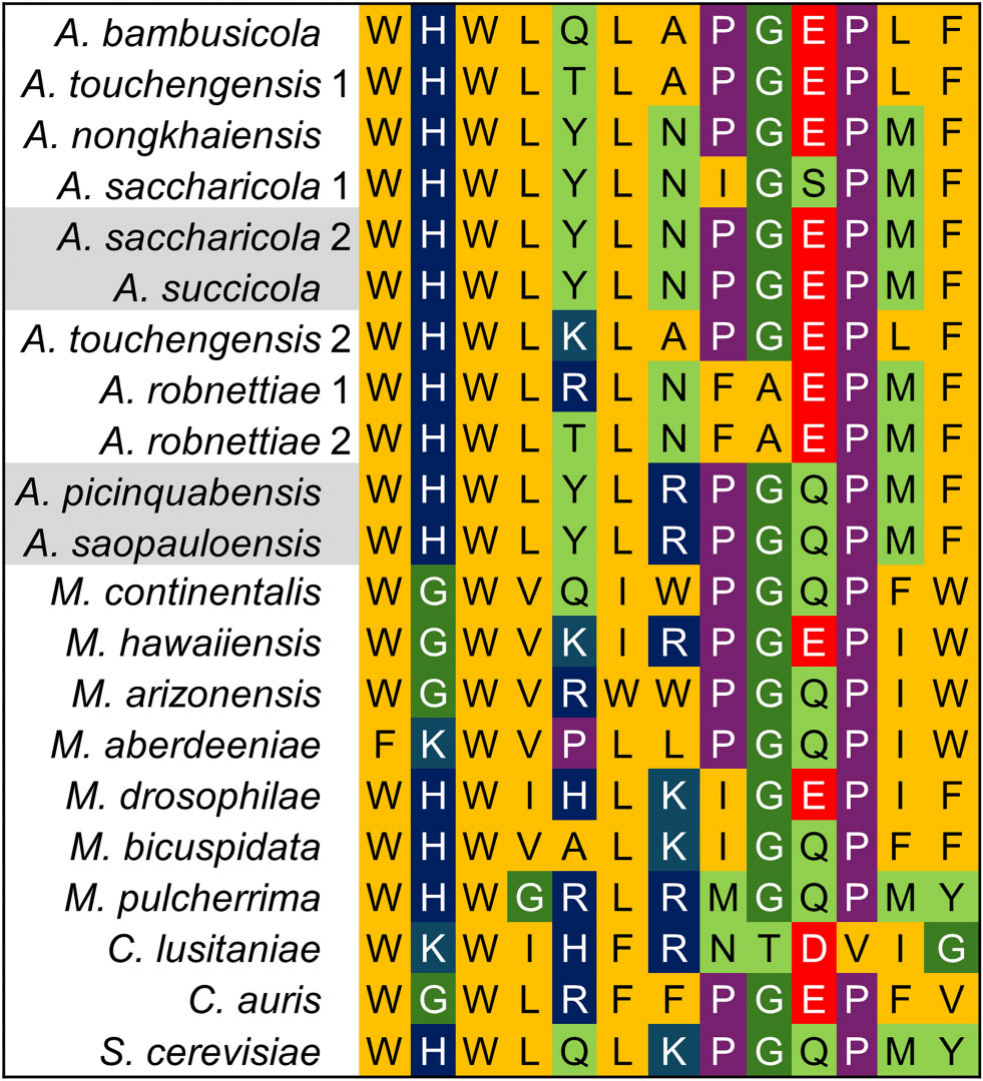
Amino acid alignment of **α**-pheromones of *Australozyma* species. Representatives of outgroup species (Lee et al. 2018) are included for comparison. A cognate peptide was not identified in the Baker2002 genome. The colours follow those generated by Geneious Prime to reflect amino acid polarity. Shading highlights identical sequences in pairs of sister species.

### Genes for host independence

Arhendt et al. (2018) identified a small number of enzymes apparently lacking in the Baker2002 genome, which would account for the inability of the yeast to grow independently from its host. It was of interest to explore this further, specifically by contrasting the state of the underlying genes in Baker2002 with that of their counterpart in free-living *Australozyma* species. The results are summarized in Table S5. Apparently functional genes accounting for eight activities found lacking in Baker2002 but present in an authentic *M. bicuspidata* were found in all free-living *Australozyma* species. The lack of function in the *Daphnia* parasite was owed in most cases to pseudogenization by early termination, although in two instances, a clear homolog could not be identified at all. The deficiency in the gene for ATP citrate synthase could not be confirmed, but this may be due to the confusing classification of the gene in question, which exists in multiple forms in *S. cerevisiae* and appears under a multitude of designations in various organisms in the grand tree of life. As to sulfur metabolism, it is worth noting that organic sulfur requirements occur in other less related yeasts, and notably in the entire genus *Saccharomycopsis*, which also shares the unique property of engaging in necrotrophic parasitism of other fungi and even some non-fungal organisms (Lachance et al. 2000). Although those species are free living, defects in sulfur acquisition might represent an early step towards the loss of growth independence.

### Asexual ascogenesis in the *Daphnia* parasite

*Australozyma monospora* sp. nov. is known exclusively, *ex hospite*, from material that contains profusions of consistently single-spored asci. The yeast can be propagated by inoculating healthy water fleas with asci recovered from infected ones. In the light of its lacking **α**-pheromone genes, in addition to other anomalies identified in the mating locus across *Australozyma* species, one would be on good grounds to hypothesize that the parasitic species has lost the ability to mate and further that is has coopted its ascus formation apparatus to produce infectious spores without the need to undergo karyogamy and meiosis. In *Metschnikowia* species, spore formation is coupled with the first meiotic division (Marinoni et al. 2003). In the absence of diploidization, which would normally follow mating of complementary mating types, a single spore would form around the single pre-mitotic haploid nucleus, and mitosis might take place inside the spore, as does the second meiotic division in *Metschnikowia* species (Lachance et al. 2024). This is consistent with the finding that reproduction in the water flea pathogen is clonal, based on an association coefficient (*I_A_*) of nearly 1 among genetic markers (Shaw et al. 2021), in sharp contrast to haplontic heterothallic *Metschnikowia* species that reproduce sexually in nature. For instance, markers in a well-sampled population of *M. hawaiiensis* had a near-zero *I_A_* value (Lachance et al. 2016), indicating that the distribution of alleles across loci was independent, as expected in a panmictic, sexually reproducing population. The formation of acerose asci with no vestiges of the conjugant cells that typically occur in haplontic *Metschnikowia* species indicates that asci of the *Daphnia* parasite arise without prior conjugation. Nor do they feature elements typical of diplontic *Metschnikowia* species, whose asci arise from the elongation of the mother cell by means of a protuberance that ranges from wide (*e.g. M. bicuspidata*), to narrow (*e.g. M. pulcherrima*), to nearly non-existent, as in *M. kunwiensis*, where the ascospore perforates the cell as it emerges (Lachance 2011). In the light of this, the report (van Uden and Castelo-Branco 1961) of long, acerose, single-spored asci in *Daphnia magna* specimens infected experimentally with *M. krissii* and *M. zobellii* is intriguing, even raising the possibility that the *Daphnia* specimens used in pathogenicity testing might already have been infected with an asexual ascogenous parasite.

### Baker2002, *Daphnia* asci, *Monospora bicuspidata, Australozyma monospora* sp. nov.

Any taxonomic disposition affecting the yeast species that infects *Daphnia* spp. will hinge on there being a robust connection between the asci observed by microscopy and the DNA sequences amplified from materials containing the asci. Barcode DNAs amplified by Wolinska et al. (2009) and Searle et al. (2015) were obtained with primers conceived by the former authors to target a single species. Although not stated unequivocally, the primers were ostensibly designed from sequences that had been amplified using universal primers, which would raise the confidence that the asci observed in the microscope are indeed the source of template DNA. The specific primers were a perfect match to Baker2002 and may not amplify DNA from other yeasts. The reason for raising this point is that amplifications using Baker2002-specific primers would yield positive results regardless of the DNA's provenance, be it the asci or free DNA present in the samples but of a different origin. This concern is justified by the fact that metabarcoding studies, of rapidly growing popularity (Harris et al. 2023), typically uncover in environmental DNA long lists of species whose presence is otherwise hardly, if at all detectable. Sequences that resemble Baker2002 barcodes have been abundantly recovered in metagenomic studies of diverse marine sediments worldwide (Lai et al. 2007, Nagano et al 2010, Thaler et al. 2012, Xu et al. 2014), raising the possibility of some kind of unexplained ubiquity. However, the sequences from sediments are sufficiently different from those amplified from *Daphnia* specimens to conclude that the two represent separate species, as D1/D2 LSU rRNA gene sequences showed 92–94% identity with those of Baker2002 (Fig. S1). Sequences of the SSU rRNA gene (97–98%, Fig. S2) and the ITS region of the rRNA gene cluster (89–90%, Fig. S3) similarly rule out the possibility that DNA amplified from *Daphnia* material originates from other ubiquitous organisms. It will be of much interest to determine the source of the DNA found in marine sediments, as it may betray the presence of a very widespread pathogenic yeast that affects another zooplankton species worldwide.

Another convincing element linking the DNA to the asci found in water fleas comes from the manner in which Arhendt et al. (2018) obtained the Baker2002 genome sequence. The material came from washed *Daphnia* specimens from which the internal microbiome was extruded. Individual parasites were repeatedly isolated by cell sorting. The curated genome completeness was high (96.7%). In the current study, none of the many individual phylogenies leading to the selection of 108 genes used to construct the species phylogeny in Fig. 1 revealed any evidence of contamination by sequences that would be better assignable to other species. There is therefore no doubt that the Baker2002 sequence in fact originates from ascospores that infected the *Daphnia* specimens examined by Arhendt et al. (2018). It does not follow, however, that the two can be unequivocally equated to Metschnikoff's (1884) *Monospora bicuspidata*. In the light of the fact that Kamienski (1899) recognized two species, *M. bicuspidata* and *M. artemiae*, a clear link, at the species level, of contemporaneous genome data to century-old drawings remains uncertain, although there is no reason to doubt that the species are congeners.

The choice of a taxonomic designation must of course comply with the dictates of the Code. Importantly, one should endeavour to minimize any collateral damage that sometimes results from the nomenclatural enterprise. The name *Monospora* cannot be used for a genus governed by the Code, but nothing prevents it from being used as epithet. The names *Monosporella* and *Metschnikowiella* have been rejected as superfluous synonyms of *Metschnikowia* (Doweld 2015). As for *Metschnikowia*, despite insuperable problems of typification, its conservation and the existence of over 60 robustly delineated species bearing that name strongly argue for the maintenance of its current use for species that form a cohesive assemblage centered around *M. bicuspidata* as typified by Wickerham (1964). Erection of the genus *Australozyma* by Liu et al. (2024) was one of the more compelling contributions of their ambitious phylogenomic analysis of the Metschnikowiaceae. The robust phylogeny presented here in Fig. 1 supports inclusion of the *Daphnia* material in that genus *via* the Baker2002 genome sequence data, a conclusion corroborated by phylogenetic analysis of barcode sequences (Figs S1, S2, S3). The existence of several publications referring to the *Daphnia* parasites as *Metschnikowia bicuspidata* might be seen as a reason at least to preserve the original epithet, which the Code would allow. In the present circumstance, however, proximity of the genera *Australozyma* and *Metschnikowia* and the impossibility of absolutely demonstrating the conspecificity of Metschnikoff's (1884) yeast with the material associated with the Baker2002 sequence would add more confusion, as the resulting binomial might be perceived as a new combination with *Metschnikowia bicuspidata* as basionym. As the author of the first Code (de Candolle 1867) admonished, the most important duty of the taxonomist is to “avoid confusion”. The binomial *Australozyma monospora*, in addition to following logically from the foregoing discussion, recognizes the historical connection of material currently observed in diseased *Daphnia* to Metschnikoff's (1884) material, without presuming their conspecificity.

### Taxonomy: *Australozyma monospora* Lachance sp. nov.

Etymology: mo.no'spo.ra, *monospora*, N.L. appositive noun, pertaining to the consistent formation of single spored asci and in recognition of Metschnikoff's excellent observations of a similar yeast species in water fleas.

Typification of an obligate parasite is problematic. The use of an illustration is allowed by Article 40.5 of the Shenzen Code (Turland 2018 et al. 2018) “if there are technical difficulties of specimen preservation or if it is impossible to preserve a specimen that would show the features attributed to the taxon by the author of the name.” The latter being possible, if not convenient, the holotype of *Australozyma monospora* sp. nov. is a microscope slide containing material cultured from the same collection that served as the source for the Baker2002 genome. The material is preserved in a metabolically inactive form (dry) and deposited in the University of Michigan Herbarium under accession number MICH 346683. It was collected from Baker Lake, Michigan, in 2002. Live isotypes are maintained in the Cáceres, Duffy, and Stewart Merrill laboratories, as well as in other laboratories that have published extensively on this host-parasite interaction (e.g. Spencer Hall and co-authors). The name is registered in MycoBank under number MB 859667.

The species has not been cultured *ex hospite*. It can be observed in infected *Daphnia dentifera* and other cladoceran species in North America, Europe, the Middle east (Halle et al. 2024) and possibly other world regions. Cells are elongate, 2 × 8 μm or longer, dividing by terminal budding on a broad base. Asci are unconjugated, acerose, 2 × 45-55 μm, and contain a single aciculate, bicuspidate ascospore, 1 × 40-50 μm. Based on the genome sequence, the species is haploid, heterothallic, and appears to differentiate into asci without undergoing a sexual cycle. The species has been characterized by whole genome sequencing of isolate Baker2002 (GCA_003 614 695, Arhendt et al. 2018) and has been identified on numerous occasions (Table S4) from barcode sequences such as FJ763559 (ITS rDNA), FJ794936 (LSU rRNA D1/D2), or FJ763541 (SSU rRNA), which are identical to those extracted from the genome sequence. Identification of any new material should rely on determining a barcode sequence.

*Australozyma* Q.M. Wang, Yurkov, Boekhout & F.Y. Bai emend. Lachance: the genus as described by Liu et al (2024) is emended to account for *A. monospora*, which does not grow in laboratory media and forms acerose asci with aciculate ascospores..

## Supplementary Material

foaf041_Supplemental_File
