# Supplementary material for: Reviving Élie Metschnikoff's Monospora: the obligately parasitic yeast Australozyma monospora sp. nov
Source: FEMS Yeast Res. 2025 Aug 5;25:foaf041. doi: 10.1093/femsyr/foaf041 (PMC12359140; doi:10.1093/femsyr/foaf041)
Supplement: foaf041_Supplemental_File [file foaf041_supplemental_file.docx]

*Supplementary data file*

**Reviving Élie Metschnikoff’s *Monospora*: the obligately parasitic yeast *Australozyma monospora* sp. nov.**

Marc-André Lachance,^1*^ Carla E. Cáceres,^2^ Molly J. Fredericks,^2^ Meghan A. Duffy,^3^ Tara E. Stewart Merrill^4^

^1^Department of Biology, University of Western Ontario, London, Ontario, Canada N6A 5B7

^2^School of Integrative Biology, University of Illinois Urbana-Champaign, Urbana, IL 61801, USA

^3^Department of Ecology and Evolutionary Biology, University of Michigan, Ann Arbor, MI 48109 USA

^4^Cary Institute of Ecosystem Studies, Millbrook, NY 12545, USA

*Corresponding author, [lachance@uwo.ca](mailto:lachance@uwo.ca)

**List of supplementary tables**

Table S1. List of genome accession numbers.

Table S2. Genes concatenated to infer a phylogeny of *Australozyma* and related Metschnikowiaceae.

Table S3. Length (kb) of detectable pairwise sequence identity surrounding mating loci of *Australozyma* species.

Table S4. Barcode sequences of uncultured fungi that match *Australozyma monospora* sp. nov.

Table S5. Genes associated with the loss of independent growth in *A. monospora* sp. nov. but present in all other *Australozyma* species.

**List of supplementary figures**

Fig S1. FastTree phylogram based on an alignment of LSU rRNA gene D1/D2 domains (327 positions) of uncultured fungi with high sequence identity to *A. monospora* sp.nov.

Fig S2. FastTree phylogram based on an alignment of SSU rRNA gene (958 positions) of uncultured fungi with high sequence identity to *A. monospora* sp. nov.

Fig S3. FastTree phylogram based on an alignment of ITS rDNA sequences (278 positions) of uncultured fungi with high sequence identity to *A. monospora* sp. nov.

Table S1. List of genome accession numbers.

| *Australozyma bambusicola* | GCA_030563705 |
| --- | --- |
| *Australozyma monospora* sp. nov. (Baker2002) | GCA_003614695 |
| *Australozyma nongkaiensis* | GCA_030563825 |
| *Australozyma picinguabensis* | GCA_030582875 |
| *Australozyma robnettiae* | GCA_030568975 |
| *Australozyma saccharicola* | GCA_030569455 |
| *Australozyma saopauloensis* | GCA_030582915 |
| *Australozyma succicola* | GCA_030563905 |
| *Australozyma touchengensis* | GCA_030566735 |
| *Candida danieliae* | GCA_030579135 |
| *Candidozyma auris* | GCA_002775015 |
| *Candidozyma haemuli* | GCA_002926055 |
| *Candidozyma ruelliae* | GCA_030582895 |
| *Clavispora lusitaniae* | GCA_001673695 |
| *Danielia oregonensis* | GCA_003707785 |
| *Debaryomyces hansenii* | GCA_000006445 |
| *Gabaldonia eppingiae* | NMDC60137102 |
| *Gaillardinia entomophila* | GCA_030555945 |
| *Helenozyma melibiosica* | GCA_030563325 |
| *Hermanozyma ubatubensis* | GCA_030567085 |
| *Metschnikowia agaves* | GCA_008065245 |
| *Metschnikowia ahupensis* | GCA_021272955 |
| *Metschnikowia amazonensis* | GCA_008065195 |
| *Metschnikowia anglica* | GCA_030573055 |
| *Metschnikowia arizonensis* | GCA_002370875 |
| *Metschnikowia australis* | GCA_002073855 |
| *Metschnikowia bicuspidata* | GCA_001664035 |
| *Metschnikowia caudata* | GCA_008065185 |
| *Metschnikowia corniflorae* | GCA_030581935 |
| *Metschnikowia drosophilae* | GCA_002893735 |
| *Metschnikowia gelsemii* | GCA_030561745 |
| *Metschnikowia gruessii* | GCA_030563445 |
| *Metschnikowia kipukae* | GCA_002370135 |
| *Metschnikowia lopburiensis* | GCA_030563105 |
| *Metschnikowia lunata* | GCA_030583235 |
| *Metschnikowia noctiluminum* | GCA_030578735 |
| *Metschnikowia orientalis* | GCA_002893685 |
| *Metschnikowia peoriensis* | GCA_030573015 |
| *Metschnikowia pimensis* | GCA_030556455 |
| *Metschnikowia proteae* | GCA_002370515 |
| *Metschnikowia pulcherrima* | GCA_030583425 |
| *Metschnikowia rubicola* | GCA_030557065 |
| *Metschnikowia* sp. yHK443 | GCA_030444905 |
| *Metschnikowia vanudenii* | GCA_030583145 |
| *Osmozyma mogii* | GCA_030573315 |
| *Osmozyma tolerans* | GCA_030582955 |
| *Sungouiella intermedia* | GCA_900106115 |
| *Tanozyma kutonensis* | GCA_030562905 |
| *Wilhelminamyces savonicus* | GCA_030570115 |

Table S2. Genes concatenated to infer a phylogeny of *Australozyma* and related Metschnikowiaceae. The placement of the Baker2002 genome (bak) relative to other species, abbreviated to the first three letters of their epithets, is shown.

| **Alignment** | |  |  |  |  |  |  |  |  |  |  |  |
| --- | --- | --- | --- | --- | --- | --- | --- | --- | --- | --- | --- | --- |
| **Median** | **1486.5** |  |  |  |  |  |  |  |  |  |  |  |
| **Gene** | **Length before editing** | **Scaffold of Baker2002 genome** | **bak(Australozyma)** | **bak(bam tou)** | **bak(pic sao)** | **Other Australozyma** | **bak kut** | **bak(epp kut)** | **bak(met rhi)** | **bak cau** | **bak yHK** | **Other outside** |
| **108** | **192310** |  | 42 | 33 | 3 | 10 | 4 | 3 | 2 | 2 | 2 | 7 |
| *ACO2* | 2063 | 1 |  |  |  |  |  | 1 |  |  |  |  |
| *ACSx* | 3924 | 8 | 1 |  |  |  |  |  |  |  |  |  |
| *ADA2* | 1359 | 9 |  | 1 |  |  |  |  |  |  |  |  |
| *ADE4* | 1630 | 8 | 1 |  |  |  |  |  |  |  |  |  |
| *AMD1* | 1898 | 2 | 1 |  |  |  |  |  |  |  |  |  |
| *AMO2* | 2090 | 2 |  |  |  |  |  |  |  | 1 |  |  |
| *APL4* | 1665 | 8 | 1 |  |  |  |  |  |  |  |  |  |
| *ARC1* | 1155 | 2 |  |  |  | 1 |  |  |  |  |  |  |
| *ARG1* | 1249 | 2 |  | 1 |  |  |  |  |  |  |  |  |
| *ARO1* | 2923 | 9 | 1 |  |  |  |  |  |  |  |  |  |
| *ARO4* | 1067 | 1 | 1 |  |  |  |  |  |  |  |  |  |
| *ATP2* | 1401 | 5 |  | 1 |  |  |  |  |  |  |  |  |
| *CAT1* | 1426 | 8 | 1 |  |  |  |  |  |  |  |  |  |
| *CCR4* | 1479 | 9 |  | 1 |  |  |  |  |  |  |  |  |
| *CCT6* | 1538 | 3 |  | 1 |  |  |  |  |  |  |  |  |
| *CHS1* | 1879 | 1 |  | 1 |  |  |  |  |  |  |  |  |
| *CHS2* | 4538 | 6 | 1 |  |  |  |  |  |  |  |  |  |
| *COQ1* | 1383 | 2 |  | 1 |  |  |  |  |  |  |  |  |
| *CPY1* | 1540 | 3 | 1 |  |  |  |  |  |  |  |  |  |
| *CRM1* | 3245 | 2 |  | 1 |  |  |  |  |  |  |  |  |
| *CRN1* | 1220 | 1 |  | 1 |  |  |  |  |  |  |  |  |
| *DHH1* | 1255 | 8 |  | 1 |  |  |  |  |  |  |  |  |
| *DRS2* | 3375 | 4 |  |  |  |  |  |  |  |  |  | 1 |
| *DUG1* | 1429 | 2 | 1 |  |  |  |  |  |  |  |  |  |
| *ENO1* | 1299 | 6 | 1 |  |  |  |  |  |  |  |  |  |
| *ERG4* | 1358 | 6 |  | 1 |  |  |  |  |  |  |  |  |
| *ETR1* | 1097 | 7 | 1 |  |  |  |  |  |  |  |  |  |
| *FAL1* | 1173 | 10 |  |  |  |  | 1 |  |  |  |  |  |
| *FAS2* | 5957 | 5 |  |  |  |  | 1 |  |  |  |  |  |
| *GAP1* | 1737 | 3 |  |  |  | 1 |  |  |  |  |  |  |
| *GDI1* | 1318 | 10 |  |  |  | 1 |  |  |  |  |  |  |
| *GEM1* | 1808 | 6 |  |  |  |  |  |  |  |  |  | 1 |
| *GLR1* | 1433 | 6 | 1 |  |  |  |  |  |  |  |  |  |
| *GTR1* | 880 | 2 |  |  |  | 1 |  |  |  |  |  |  |
| *HATx* | 1962 | 8 |  |  |  | 1 |  |  |  |  |  |  |
| *HDA1* | 1114 | 2 |  | 1 |  |  |  |  |  |  |  |  |
| *HNM1* | 1486 | 7 | 1 |  |  |  |  |  |  |  |  |  |
| *HOS2* | 1188 | 6 |  | 1 |  |  |  |  |  |  |  |  |
| *HSP78* | 2118 | 2 |  | 1 |  |  |  |  |  |  |  |  |
| *ICL1* | 1634 | 5 |  | 1 |  |  |  |  |  |  |  |  |
| *ILV5* | 927 | 6 |  |  |  |  |  |  |  |  |  | 1 |
| *IMPx* | 1623 | 9 | 1 |  |  |  |  |  |  |  |  |  |
| *KAR2* | 1969 | 2 |  | 1 |  |  |  |  |  |  |  |  |
| *KFG57* | 4860 | 5 |  | 1 |  |  |  |  |  |  |  |  |
| *KGD1* | 2106 | 3 |  |  |  |  |  |  |  |  | 1 |  |
| *LCB2* | 1422 | 10 |  |  |  | 1 |  |  |  |  |  |  |
| *LETM1x* | 1064 | 9 |  | 1 |  |  |  |  |  |  |  |  |
| *LEU1* | 1923 | 8 | 1 |  |  |  |  |  |  |  |  |  |
| *LPD1* | 1441 | 10 | 1 |  |  |  |  |  |  |  |  |  |
| *LYS12* | 928 | 2 |  | 1 |  |  |  |  |  |  |  |  |
| *LYS9* | 1312 | 3 |  |  |  |  |  |  | 1 |  |  |  |
| *MDH1* | 984 | 2 |  | 1 |  |  |  |  |  |  |  |  |
| *MDM31* | 1386 | 6 | 1 |  |  |  |  |  |  |  |  |  |
| *MDN1* | 2700 | 9 |  |  |  |  | 1 |  |  |  |  |  |
| *MFSx* | 1395 | 8 |  |  |  | 1 |  |  |  |  |  |  |
| *MGM1* | 2081 | 10 | 1 |  |  |  |  |  |  |  |  |  |
| *MITx* | 1774 | 7 | 1 |  |  |  |  |  |  |  |  |  |
| *MLS1* | 1653 | 6 |  | 1 |  |  |  |  |  |  |  |  |
| *MSB3* | 1393 | 10 |  | 1 |  |  |  |  |  |  |  |  |
| *MSH2* | 969 | 4 |  | 1 |  |  |  |  |  |  |  |  |
| *MSH6* | 2865 | 9 | 1 |  |  |  |  |  |  |  |  |  |
| *MTR4* | 2106 | 3 |  |  |  |  |  |  |  |  |  | 1 |
| *NOP58* | 1303 | 3 |  | 1 |  |  |  |  |  |  |  |  |
| *OLA* | 1191 | 4 |  |  |  |  |  |  |  |  |  | 1 |
| *PAB1* | 1649 | 5 |  | 1 |  |  |  |  |  |  |  |  |
| *PEL1* | 1630 | 10 | 1 |  |  |  |  |  |  |  |  |  |
| *PFS2* | 1108 | 4 | 1 |  |  |  |  |  |  |  |  |  |
| *PHO8* | 1369 | 8 | 1 |  |  |  |  |  |  |  |  |  |
| *PKA1* | 977 | 1 |  |  |  | 1 |  |  |  |  |  |  |
| *PKC1* | 1012 | 2 |  | 1 |  |  |  |  |  |  |  |  |
| *PMA1* | 2532 | 6 | 1 |  |  |  |  |  |  |  |  |  |
| *POB3* | 1532 | 5 |  |  |  | 1 |  |  |  |  |  |  |
| *PRT1* | 2061 | 6 | 1 |  |  |  |  |  |  |  |  |  |
| *PSPx* | 1453 | 8 |  |  | 1 |  |  |  |  |  |  |  |
| *PXA2* | 1883 | 1 | 1 |  |  |  |  |  |  |  |  |  |
| *PYC1* | 3500 | 3 |  |  |  |  |  | 1 |  |  |  |  |
| *RCY1* | 1660 | 63 |  |  | 1 |  |  |  |  |  |  |  |
| *RPL48* | 1107 | 2 | 1 |  |  |  |  |  |  |  |  |  |
| *RPN2* | 2675 | 2 |  | 1 |  |  |  |  |  |  |  |  |
| *RTP6* | 1181 | 3 |  |  |  |  |  |  | 1 |  |  |  |
| *RVB1* | 1373 | 3 |  | 1 |  |  |  |  |  |  |  |  |
| *SCH9* | 1391 | 3 | 1 |  |  |  |  |  |  |  |  |  |
| *SDH1* | 1872 | 1 | 1 |  |  |  |  |  |  |  |  |  |
| *SEC2* | 1685 | 4 | 1 |  |  |  |  |  |  |  |  |  |
| *SER33* | 1274 | 2 |  |  | 1 |  |  |  |  |  |  |  |
| *SES1* | 1360 | 10 |  |  |  |  |  |  |  |  |  | 1 |
| *SHM1* | 1409 | 3 |  | 1 |  |  |  |  |  |  |  |  |
| *SIN3* | 1854 | 6 |  |  |  |  | 1 |  |  |  |  |  |
| *SMTx* | 1487 | 6 | 1 |  |  |  |  |  |  |  |  |  |
| *SNF2* | 2517 | 8 | 1 |  |  |  |  |  |  |  |  |  |
| *STH1* | 2755 | 3 |  | 1 |  |  |  |  |  |  |  |  |
| *STI1* | 1927 | 10 |  |  |  | 1 |  |  |  |  |  |  |
| *TEF3* | 3163 | 2 |  | 1 |  |  |  |  |  |  |  |  |
| *THS1* | 2069 | 2 |  | 1 |  |  |  |  |  |  |  |  |
| *TIM4* | 1210 | 7 | 1 |  |  |  |  |  |  |  |  |  |
| *TPK2* | 1006 | 2 | 1 |  |  |  |  |  |  |  |  |  |
| *TUB1* | 1295 | 4 |  |  |  |  |  |  |  | 1 |  |  |
| *TUB2* | 1257 | 6 |  | 1 |  |  |  |  |  |  |  |  |
| *UGA1* | 1351 | 6 | 1 |  |  |  |  |  |  |  |  |  |
| *UPG1* | 1462 | 9 | 1 |  |  |  |  |  |  |  |  |  |
| *URA9* | 1180 | 4 | 1 |  |  |  |  |  |  |  |  |  |
| *VAC8* | 1542 | 7 |  |  |  |  |  |  |  |  | 1 |  |
| *VMA2* | 1464 | 1 |  |  |  |  |  | 1 |  |  |  |  |
| *VPH1* | 2141 | 7 | 1 |  |  |  |  |  |  |  |  |  |
| *VPS1* | 2032 | 7 | 1 |  |  |  |  |  |  |  |  |  |
| *YAT1* | 1631 | 8 | 1 |  |  |  |  |  |  |  |  |  |
| *YCF1* | 3833 | 3 | 1 |  |  |  |  |  |  |  |  |  |
| *YME1* | 1773 | 4 |  |  |  |  |  |  |  |  |  | 1 |

Table S3. Length (kb) of detectable pairwise sequence identity surrounding mating loci of *Australozyma* species.

| sac |  |  |  |  |  |  |  |  |  |
| --- | --- | --- | --- | --- | --- | --- | --- | --- | --- |
| 99.8 | suc |  |  |  |  |  |  |  |  |
| 97.7 | 217.6 | non |  |  |  |  |  |  |  |
| 22.0 | 43.3 | 7.7 | rob |  |  |  |  |  |  |
| 72.0 | 74.4 | 72.0 | 4.3 | sao |  |  |  |  |  |
| 71.0 | 7.9 | 71.2 | 7.4 | 87.0 | pic |  |  |  |  |
| 6.2 | 13.8 | 10.9 | 6.3 | 6.3 | 3.5 | bam |  |  |  |
| 1.4 | 6.0 | 6.3 | 6.5 | 6.1 | 6.2 | 27.5 | tou |  |  |
| 1.3 | 8.0 | 7.1 | 8.1 | 7.9 | 7.7 | 1.2 | 6.8 | bak |  |
| 8.4 | 9.0 | 7.5 | 7.4 | 8.3 | 7.5 | 6.3 | 6.5 | 7.9 | Length of locus |

Table S4. Barcode sequences of uncultured fungi that match *Australozyma monospora* sp. nov.

| **ITS** | | | | | **LSU D1/D2** | | **SSU** | |
| --- | --- | --- | --- | --- | --- | --- | --- | --- |
| AB507820 | AB615474 | AB615505 | AB615528 | FJ763565 | FJ794936 | ON411380 | AB275102 | FJ763556 |
| AB507821 | AB615475 | AB615506 | AB615529 | FJ763566 | FJ794937 | ON411595 | AB468630 | FJ763557 |
| AB507822 | AB615477 | AB615507 | AB615534 | FJ763567 | FJ794938 | ON411596 | AB468634 | KF658207 |
| AB507823 | AB615478 | AB615509 | AB615537 | FJ763568 | FJ794939 | ON411597 | AB468676 | KF658209 |
| AB507836 | AB615479 | AB615510 | AB615538 | FJ763569 | FJ794940 | ON411598 | AB514601 | KF658210 |
| AB507837 | AB615480 | AB615511 | AB615539 | FJ763570 | FJ794941 |  | FJ763540 | KF658211 |
| AB507839 | AB615482 | AB615512 | AB615541 | FJ763571 | FJ794942 |  | FJ763541 | KF658212 |
| AB507842 | AB615483 | AB615513 | AB615543 | FJ763572 | FJ794943 |  | FJ763542 | KF658213 |
| AB507843 | AB615485 | AB615514 | AB615544 | GU117070 | JF821198 |  | FJ763543 | KF658216 |
| AB507848 | AB615486 | AB615515 | AB615545 | KF658196 | JF821200 |  | FJ763544 |  |
| AB507853 | AB615487 | AB615516 | AB615570 | KF658197 | JF821205 |  | FJ763545 |  |
| AB615457 | AB615490 | AB615517 | AB615572 | KF658198 | JF821207 |  | FJ763546 |  |
| AB615458 | AB615491 | AB615518 | AB615573 | KF658199 | JF821208 |  | FJ763547 |  |
| AB615461 | AB615492 | AB615519 | DQ279844 | KF658201 | JF821209 |  | FJ763548 |  |
| AB615463 | AB615493 | AB615520 | FJ763558 | KF658202 | JF821210 |  | FJ763549 |  |
| AB615464 | AB615494 | AB615522 | FJ763559 | KF658205 | JF821211 |  | FJ763550 |  |
| AB615467 | AB615495 | AB615523 | FJ763560 | KF658206 | JF821212 |  | FJ763551 |  |
| AB615468 | AB615497 | AB615524 | FJ763561 | KJ194387 | JF821213 |  | FJ763552 |  |
| AB615471 | AB615499 | AB615525 | FJ763562 | KT758085 | JF821214 |  | FJ763553 |  |
| AB615472 | AB615500 | AB615526 | FJ763563 | KT758107 | ON411378 |  | FJ763554 |  |
| AB615473 | AB615501 | AB615527 | FJ763564 | KT758137 | ON411379 |  | FJ763555 |  |

Table S5. Genes associated with the loss of independent growth in *A. monospora* *sp. nov.* but present in all other *Australozyma* species. The missing enzymes were identified by Ahrendt et al. (2018) in the Baker2002 genome. Corresponding genes were identified for *S. cerevisiae* in the SGD database and queried against *Australozyma* genomes. The reason for the absence of functionality in *A. monospora* sp. nov. is given. Position of last amino acid is given for nonsense mutations.

| Enzyme | Gene | Protein size | | *A. monospora* sp. nov. |
| --- | --- | --- | --- | --- |
|  |  | *Australozyma* | *S. cerevisiae* |  |
| ATP sulfurylase | *MET3* | 523-524 | 512 | No homolog |
| APS kinase | *MET14* | 200 | 203 | Stop at 11 |
| PAPS reductase | *MET16* | 248 | 262 | Stop at 129 |
| Sulfite reductase α | *MET10* | 1111-1125 | 1036 | Stop at 32 |
| Sulfite reductase β | *MET5* | 1417-1427 | 1443 | Low identity, no start |
| Hydroxymethyl pyrimidine kinase* | *THI20*  *THI21* | 574-591 | 552  552 | Stop at 157, no start |
| Thiamine phosphate synthase | *THI4* | 331-333 | 327 | No clear homolog |
| Hydroxyethyl thiazole kinase | *THI6* | 509-525 | 541 | Stop at 3, no start |

*Only one homolog was found in each *Australozyma* species. In *S. cerevisiae*, the two proteins differ at 77 amino acid positions.


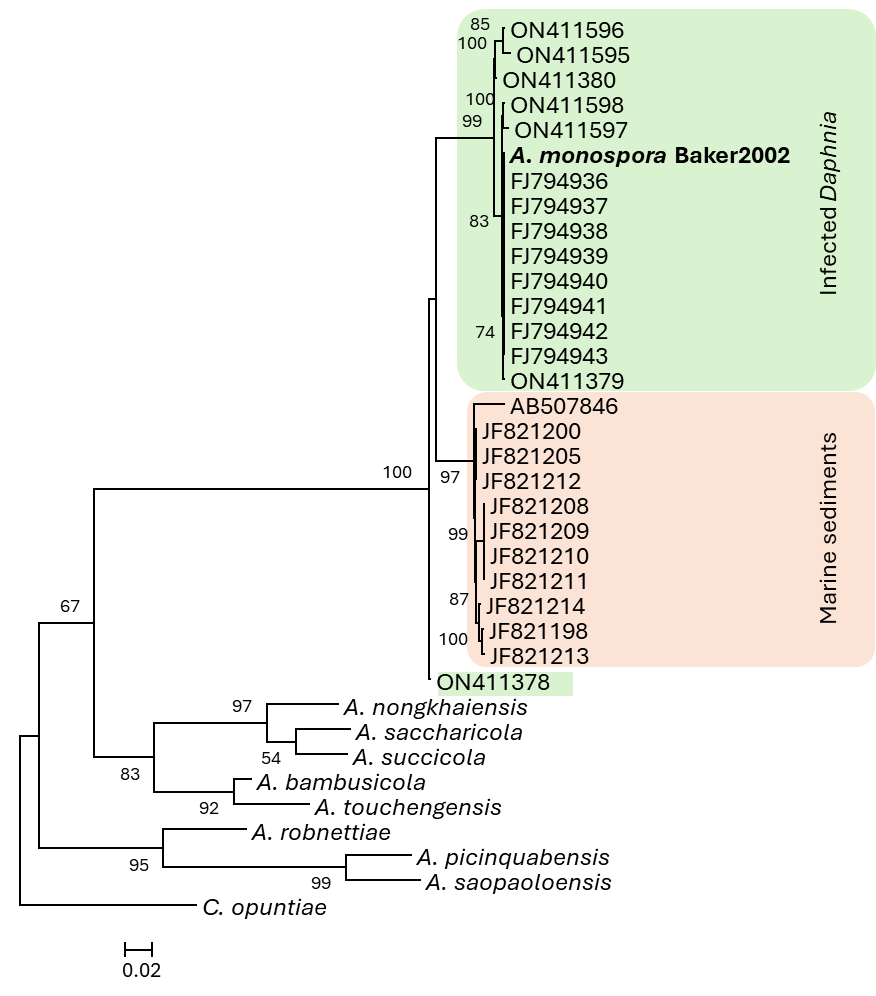


Fig S1. FastTree phylogram based on an alignment of LSU rRNA gene D1/D2 domains (327 positions) of uncultured fungi with high sequence identity to *A. monospora* sp. nov. Other *Austalozyma* species are included for comparison, with *Clavispora opuntiae* as outgroup to position the root. Shimodaira-Hasegawa clade support values of 50% or more are shown. Sequences are associated with *Daphnia* spp. (Wolinska et al. 2009, FJ; Shaw et al. 2022 unpublished, ON) or marine sediments (Nagano et al. 2010, AB; Thaler et al. 2012, JF).


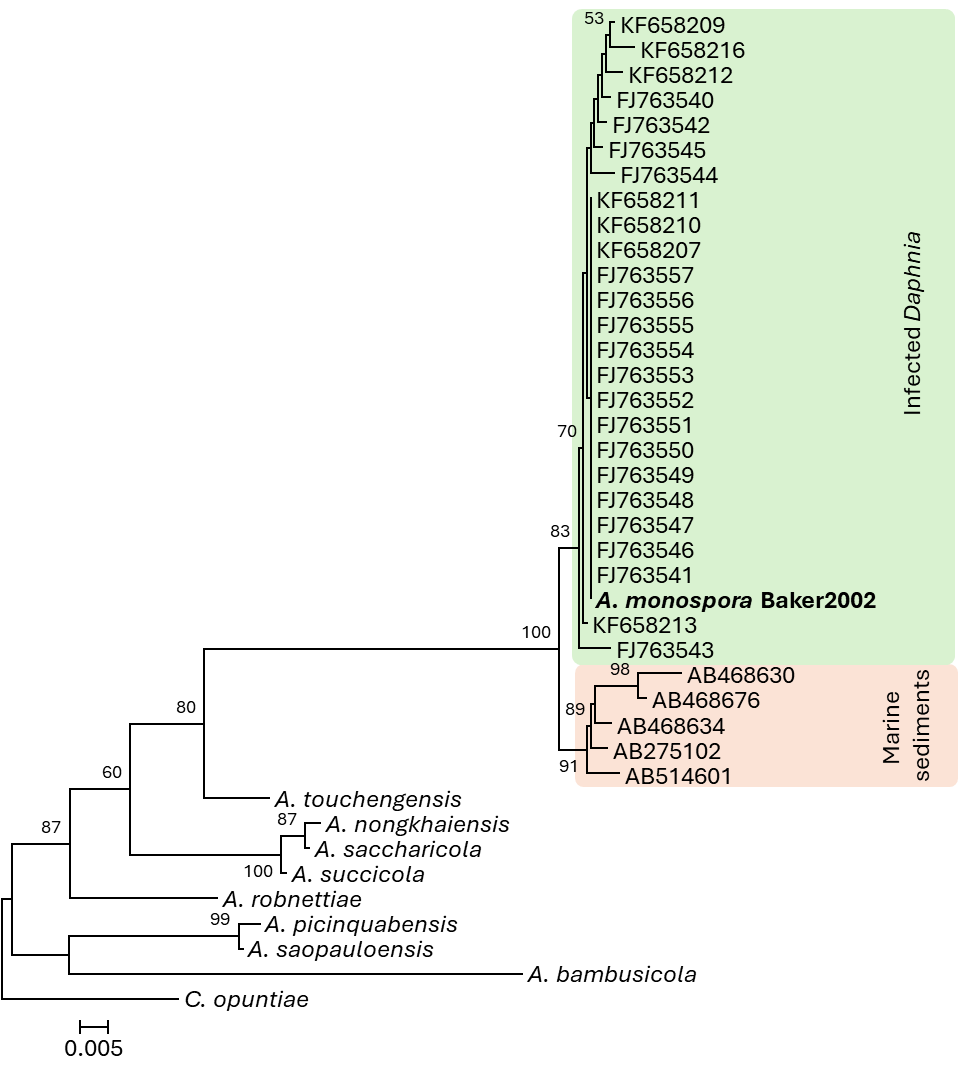


Fig S2. FastTree phylogram based on an alignment of SSU rRNA gene (958 positions) of uncultured fungi with high sequence identity to *A. monospora* sp. nov. Other *Austalozyma* species are included for comparison, with *Clavispora opuntiae* as outgroup to position the root. Shimodaira-Hasegawa clade support values of 50% or more are shown. Sequences are associated with *Daphnia* spp. (Wolinska et al. 2009, FJ; Searle et al. 2015, KF), or marine sediments (Nagano et al. 2010, AB).


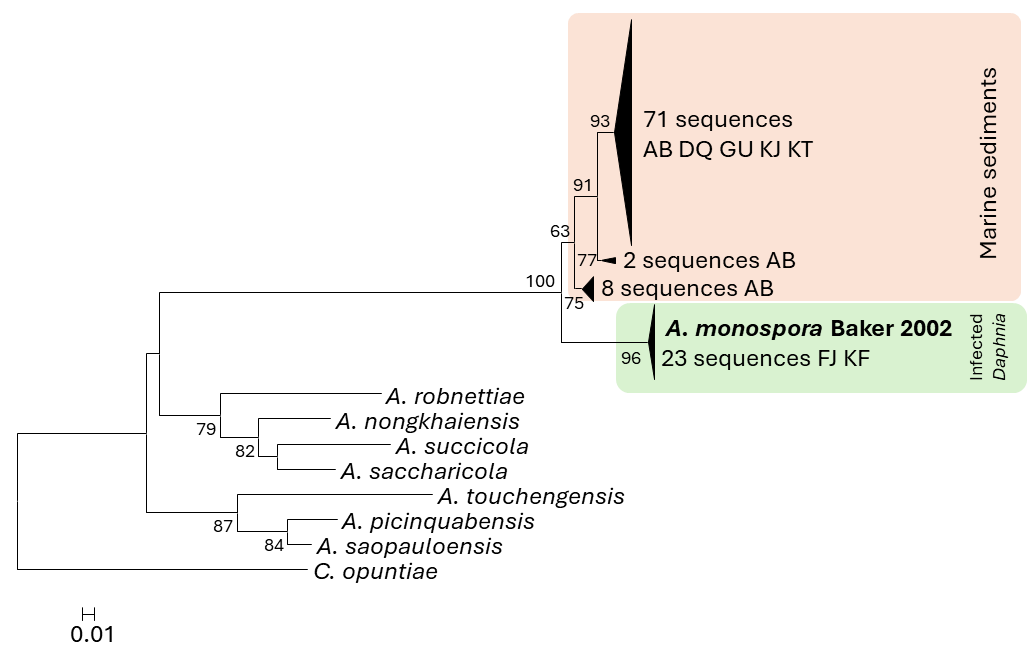


Fig S3. FastTree phylogram based on an alignment of ITS rDNA sequences (278 positions) of uncultured fungi with high sequence identity to *A. monospora* sp. nov. Other *Austalozyma* species are included for comparison, with *Clavispora opuntiae* as outgroup to position the root. Shimodaira-Hasegawa clade support values of 50% or more are shown. Sequences are associated with *Daphnia* spp. (Wolinska et al., 2009, FJ; Searle et al. 2015, KF) or marine sediments (Nagano et al. 2010, AB; Lai et al. 2007, DQ; Burgaud et al. 2009 unpublished, GU; Xu et al. 2014, KJ, KT).
